# Robust Variable Selection with Exponential Squared Loss for the Spatial Durbin Model

**DOI:** 10.3390/e25020249

**Published:** 2023-01-30

**Authors:** Zhongyang Liu, Yunquan Song, Yi Cheng

**Affiliations:** College of Science, China University of Petroleum, Qingdao 266580, China

**Keywords:** spatial Durbin model, exponential squared loss, robust variable selection

## Abstract

With the continuous application of spatial dependent data in various fields, spatial econometric models have attracted more and more attention. In this paper, a robust variable selection method based on exponential squared loss and adaptive lasso is proposed for the spatial Durbin model. Under mild conditions, we establish the asymptotic and “Oracle” properties of the proposed estimator. However, in model solving, nonconvex and nondifferentiable programming problems bring challenges to solving algorithms. To solve this problem effectively, we design a BCD algorithm and give a DC decomposition of the exponential squared loss. Numerical simulation results show that the method is more robust and accurate than existing variable selection methods when noise is present. In addition, we also apply the model to the 1978 housing price dataset in the Baltimore area.

## 1. Introduction

In recent years, spatial section data have been widely used in geography, politics, environment, and other fields. Therefore, spatial econometrics, a model initially used in the economic area, has also attracted much attention. Anselin (1988) [1] divides spatial econometric models into spatial error models, spatial hysteresis models, and spatial Durbin models (SDM). Among them, the spatial Durbin model is represented as y=ρWy+Xβ+WXδ+ε. The spatial Dubin model considers the influence of the independent variable and the dependent variable of the spatial lag on the dependent variable simultaneously and can more easily estimate the unbiased coefficient. At the same time, the spatial Dubin model can also calculate spatial spillover effects based on panel data. In spatial regression analysis, the influence of regional locations on observations is expressed employing spatial weight matrix *W*, and the appropriate setting of spatial weight matrix is an essential basis for spatial econometric analysis. There are two main ways to select a spatial weight matrix: The first method is to select a spatial weight matrix from an optional set of spatial weight matrices. Kelejian (2008) [2] uses GMM estimation to select an actual spatial weight matrix. A non-nested J test method is proposed to test a set of alternative models with different spatial weight matrices for the empty SAR model. The second type of method estimates the weight matrix by averaging different spatial weight matrices. Zhang and Yu (2018) [3] propose a model averaging process to reduce estimation error. This approach overcomes the difficulty that the actual spatial weight matrix is not in the candidate matrix.

In the field of classical linear regression, much work has contributed to variable selection. Among them, the most popular method is to add penalty functions to the model for variable selection. These punishment regression methods have a unified theoretical framework, such as most minor absolute shrinkage and selection operators (lasso, Tibshirani, 1996) [4], smoothly clipped absolute deviation (SCAD, Fan and Li, 2001) [5], and adaptive lasso (Zou, 2006) [6]. Since SDM has spatial autocorrelation, the above variable selection method can be directly applied to the SDM model.

Due to noise and outliers, the classical variable selection methods in regression models often face the problem of instability, so many scholars have proposed some robust variable selection algorithms. The Huber loss function was widely used in early studies, but this function has some limitations in efficiency and solution. Wang et al. (2013) [7] proposed a robust parameter estimation method based on the exponential squared loss function, which is widely used in boosting algorithms (Friedman et al., 2000) [8]. When γ is small, the loss of experience caused by a larger |t| value is close to 1; therefore, the loss function is robust to parameter estimation. Wang et al., (2013) [7] also point out that this method is more robust than other robust variable selection methodsm such as Huber estimation, quantile regression estimation (Koenker and Bassett, 1978) [9], and compound quantile regression estimation (Zou and Yuan, 2008) [10], and proposed the selection method of parameter γ.

Our research focuses on variable selection for spatial Durbin models. The spatial Durbin model combines the spatial interaction of dependent and explanatory variables, but only a few researchers use and study this model. Beer and Rield (2011) [11] used the maximum likelihood estimation to estimate the parameters of the spatial Durbin model. They used the Monte Carlo method to analyze the characteristics of the estimator. Mustaqim (2018) [12] discusses instrumental variable efficiency in simultaneous spatial Durbin models. Estimation methods are 2SLS and GMM-S2SLS. The analysis results show that the GMM-S2SLS method produces less bias than the 2SLS method. Zhu, Yanli (2020) [13] proposed parameter estimation of the spatial Durbin model based on Markov Chain Monte Carlo (MCMC). Wei, Lili (2021) [14] proposed a within-group spatial two-stage least squares estimator. However, the existing variable selection methods are affected by outliers in limited samples and are not robust enough. Therefore, it is imperative to study a robust variable selection method.

Considering robustness, we combine parameter penalty with exponential square loss and assume that the errors of the model are independent and identically distributed. For the parameter penalty method, we use adaptive lasso. We applied the robust selection method based on the exponential squared loss variable to the spatial autoregressive model and achieved satisfactory results [15]. The spatial autoregressive model is one of the special cases of the spatial Durbin model. In this paper, we aim to study the application of the robust selection method based on the exponential squared loss variable in the spatial Durbin model.

A robust variable selection method for the spatial Durbin model based on adaptive lasso penalty and exponential square loss function is proposed in this paper. This method cannot only estimate the regression coefficient but also has the function of variable selection. Next, we show the framework of the paper.

1. We build a robust variable selection method for SDM, equipped with an exponential squared loss, resistant to the influence of outliers in the observed values and errors estimating the space weight matrix.
2. To solve the optimization problem of SDM, we propose a block coordinate descent (BCD) algorithm. Secondly, to solve the subproblems generated by the BCD algorithm, we design the DC decomposition of exponential square loss and construct the CCCP program. Finally, to obtain the BCD algorithm’s convergence, we analyze the algorithm’s convergence rate to the stagnation point under mild conditions.
3. We proved the “Oracle” property of the robust variable selection method and conducted numerical experiments to verify the robustness and effectiveness of the model. Numerical studies show that when there are outliers in the observed data, the method proposed in this paper is superior to the comparison method in correctly identifying zero coefficients, nonzero coefficients, and MedSE incorrectly.

The structure of this paper is as follows. Section 2 introduces the spatial Durbin model and gives the exponential square loss function based on adaptive lasso. In Section 3, we propose an effective algorithm to complete the variable selection process. In order to check the performance of the model under limited samples, we have carried out a numerical simulation in Section 4. In Section 5, we apply our model to real-world datasets. We summarize the full text in Section 6.

## 2. Variable Selection and Estimation

### 2.1. Spatial Durbin Model

The observed dependent variable $Y_{i}\in R^{1\times 1}$, and the corresponding independent vector $X_{i}=\left(X_{i1},\ldots ,X_{ip}\right)$, where the *p* is a fixed constant. Let the dependent variable vector $Y={\left(Y_{1},\ldots ,Y_{n}\right)}^{T}$ and the independent variable matrix $X={\left(X_{1},\ldots ,X_{n}\right)}^{T}\in R^{n\times p}$. The spatial Durbin model is as follows:

(1) $$\begin{array}{c} Y=\rho WY+X\beta +WX\delta +\varepsilon . \end{array}$$

where the regression coefficient vector $\beta ={\left(\beta_{1},\ldots ,\beta_{p}\right)}^{T}\in R^{p\times 1}$, the spatial autocorrelation coefficient $\rho \in R^{1\times 1}$, the regression coefficient vector of exogenous variables $\delta ={\left(\delta_{1},\ldots ,\delta_{p}\right)}^{T}\in R^{p\times 1}$, and the error vector $\varepsilon ={\left(\varepsilon_{1},\ldots ,\varepsilon_{n}\right)}^{T}\in R^{n\times 1}$. WX is a spatial lag term that reflects the interaction of independent variables between individuals. Wy embodies the interaction between the strain variable *y* and its surrounding *y*. We assume that noises ε all obey $N\left(0,\sigma^{2}\right)$ and are independent of each other. y can be expressed as the following formula:

(2) $$\begin{array}{c} Y={\left(I_{n}-\rho W\right)}^{-1}\left(X\beta +WX\delta +\varepsilon \right). \end{array}$$

Since the maximum eigenvalue of *W* is 1 after normalization, to guarantee $\left(I_{n}-\rho W\right)$ reversibility, we order |ρ|<1. Additionally, in this article, we ignore the endogenous nature of the model.

### 2.2. Variable Selection Method for SDM

Rewrite model (1) as model (3) in the following form:

(3) $$\begin{array}{c} \varepsilon_{i}\left(\theta \right)=Y_{i}-\left(\rho WY_{i}+X_{i}\beta +WX_{i}\delta \right). \end{array}$$

Take the variable selection for the SDM into consideration. In practical applications, the regression coefficient vector $\beta^{*}$ is usually sparse. At the same time, sparse solutions can find useful dimensions and reduce redundancy, as well as improve the accuracy and robustness of regression prediction (Fan and Li, 2001 [5]; Tibshirani, 1996 [4]). Applying the penalized method to variable selection is natural, which can select essential variables and estimate the regression coefficient. In this article, we punish the loss function using the adaptive lasso penalty function. The adaptive lasso penalty is described as follows:

(4) $$\sum_{j=1}^{p}P\left(\left|\beta_{j}\right|\right)=\sum_{j=1}^{p}\eta_{j}\left|\beta_{j}\right|.$$

where $\eta_{j}=\frac{1}{|\hat{\beta_{j}}|}$, $\hat{\beta_{j}}$ is generally given by least squares estimates. Considering that the exponential square loss function has good robustness, we use it as the model’s loss function in this paper. The exponential square loss expression is as follows:

(5) $$\phi_{\gamma }\left(t\right)=1-\exp\left(-t^{2}/\gamma \right).$$

Here, γ is a parameter that controls the robustness of the loss function. γ limits the effect of outliers on the model but also reduces the accuracy of the model. Therefore, it is essential to choose the right γ. The method of selecting the right γ is shown in Section 2.4.

The model is constructed on the basis of the above model (3). The objective function to be solved is as follows:

(6) $${{\min_{\beta \in R^{p},\delta \in R^{p},\rho \in \left[0,1\right]}L\left(\beta ,\delta ,\rho \right)=\frac{1}{n}\sum_{i=1}^{n}\phi_{\gamma }\left(Y_{i}-\rho Y_{i}-X_{i}\beta -WX_{i}\delta \right)+\lambda \sum_{j=1}^{p}\eta_{j}|\beta }_{j}\left|+\lambda \sum_{j=1}^{p}\sigma_{j}\right|\delta }_{j}|.$$

We may as well order

$$\tilde{Y}=WY,$$

$$\tilde{X}=\left[X,WX\right],$$

$$\tilde{\beta }={\left[\beta^{T},\delta^{T}\right]}^{T}.$$

We can obtain a simplified expression of (6) as follows as (7):

(7) $$\min_{\tilde{\beta }\in R^{2p},\rho \in \left[0,1\right]}L\left(\tilde{\beta },\rho \right)=\sum_{i=1}^{n}\phi_{\gamma }\left(Y_{i}-\rho {\tilde{Y}}_{i}-{\tilde{X}}_{i}\tilde{\beta }\right)+\lambda \sum_{j=1}^{2p}\eta_{j}\left|{\tilde{\beta }}_{j}\right|,$$

where λ>0 is a regularization parameter. $\phi_{\gamma }\left(.\right)$ is exponential squared loss.

### 2.3. Oracle Properties and Large Sample Properties

In this section, we discuss the large sample properties and oracle properties of the proposed spatial Durbin model parameter estimation method.

First of all, let us make the true value of $\tilde{\beta }\;\mathrm{be}\;{\tilde{\beta }}_{0}={\left({\tilde{\beta }}_{10},\ldots ,{\tilde{\beta }}_{2p0}\right)}^{T}$. Additionally, because ${\tilde{\beta }}_{0}={\left[{\beta_{0}}^{T},{\delta_{0}}^{T}\right]}^{T}$, where $\beta_{0}={\left({\beta_{10}}^{T},{\beta_{20}}^{T}\right)}^{T}$, $\delta_{0}={\left({\delta_{10}}^{T},{\delta_{20}}^{T}\right)}^{T}$. Based on the sparsity assumed above, we assume that $\beta_{20}=0,\delta_{20}=0$. So, ${\tilde{\beta }}_{0}={\left[{\beta_{10}}^{T},0^{T},{\delta_{10}}^{T},0^{T}\right]}^{T}$. For the convenience of expression, we make a transformation to the ${\tilde{\beta }}_{0}$, so that ${\tilde{\beta }}_{0}={\left[{\beta_{10}}^{T},{\delta_{10}}^{T},0^{T},0^{T}\right]}^{T}={\left[{{\tilde{\beta }{}^{\prime }}_{10}}^{T},0^{T}\right]}^{T}$. In order to adapt to this transformation in ${\tilde{\beta }}_{0}$, $\tilde{X}$ needs to make a similar transformation. In the following, we all assume that $\tilde{X}$ was transformed accordingly. For convenience, we express ${\tilde{\beta }{}^{\prime }}_{10}$ as ${\tilde{\beta }}_{10}$ in the following text. Let $\hat{\tilde{\beta }}={\left({\hat{\tilde{\beta }}}_{1}^{T},{\hat{\tilde{\beta }}}_{2}^{T}\right)}^{T}$ be the resulting estimator of (4), suppose that the $\hat{\tilde{\beta }}$ here has also undergone the above transformation. $I\left(\tilde{\beta },\gamma \right)=\frac{2}{\gamma }\int ZZ^{T}e^{-r^{2}/\gamma }\left(\frac{2r^{2}}{\gamma }-1\right)dF\left(Z,y\right)$, where r=Y−${\left(I_{n}-\rho W\right)}^{-1}\tilde{X}\tilde{\beta }=Y-Z\tilde{\beta },Z={\left(I_{n}-\rho W\right)}^{-1}\tilde{X},a_{n}=\max\left\{p_{\lambda_{nj}}^{\prime }\left(\left|{\tilde{\beta }}_{0j}\right|\right):{\tilde{\beta }}_{0j}\neq 0\right\},b_{n}=\max\left\{p_{\lambda_{nj}}^{\prime \prime }\left(\left|{\tilde{\beta }}_{0j}\right|\right):{\tilde{\beta }}_{0j}\neq 0\right\}$. Let the true value of ρ be $\rho_{0}$. Thus, $\theta_{0}=\left(\rho_{0},{\tilde{\beta }}_{0}\right)$. For ease of presentation, let ${\tilde{\beta }}_{10}=\rho$ and ${\tilde{\beta }}_{1j}={\tilde{\beta }}_{1j},j=1,2,\ldots ,s$, then denote ${\tilde{\beta }}_{1}={\left(\rho ,{\tilde{\beta }}_{11},\ldots ,{\tilde{\beta }}_{1s}\right)}^{T}$ and ${\tilde{\beta }}_{01}={\left(\rho_{0},{\tilde{\beta }}_{01},\ldots ,{\tilde{\beta }}_{0s}\right)}^{T}$.

We prove the asymptotic and oracle properties of the proposed penalty estimators. Before we can prove it, we need the following hypothesis.

**Assumption 1.**  *$\Sigma =E\left(ZZ^{T}\right)$ is positive definite and ${E∥Z∥}^{3}<\infty$.*

**Assumption 2.**  *The matrix $I_{n}-\rho W$ is nonsingular with |ρ|<1*.

**Assumption 3.**  *The row and column sums of the matrices $W_{n}$ and $I-\rho W_{n}$ are bounded uniformly in absolute value.*

**Assumption 4.**  *For matrix $G_{n}=W{\left(I-\rho W\right)}^{-1}$, there exists a constant ${\tilde{\lambda }}_{c}$ such that ${\tilde{\lambda }}_{c}I_{n}-G_{n}G_{n}^{T}$ is positive semidefinite for all n*.

**Assumption 5.**  *$1/\min_{s+1\leq j\leq p}\lambda_{j}=o_{p}$ (1). Additionally, with probability 1,*

${lim inf}_{n\to \infty }\lim\inf_{t\to 0^{+}}\left\{\min_{s+1\leq j\leq p}\frac{p_{\lambda_{j}\left(t\right)}^{\prime }}{\lambda_{j}}\right\}>0$.

**Assumption 6.**  *$\sqrt{n}a_{n}=o_{p}\left(1\right),b_{n}=o_{P}\left(1\right)$.*

**Assumption 7.**  *$\left(\gamma_{n}-\gamma_{0}\right)=o_{p}\left(1\right)$ for some $\gamma_{0}>0$.*

**Assumption 8.**  *There are constants $C_{1}$ and $C_{2}$ such that, when $\theta_{1},\theta_{2}>C_{1}\lambda_{j}\left|p_{\lambda_{j}}^{\prime \prime }\left(\theta_{1}\right)-p_{\lambda_{j}}^{\prime \prime }\left(\theta_{2}\right)\right|\leq C_{2}\left|\theta_{1}-\theta_{2}\right|$, for j=0,1,…,p*.

For our proposed estimator, we give the following sample properties. The following theorem gives the consistency and “oracle" property of the proposed estimator.

**Theorem 1.**  *If Assumptions 1−8 are true, then there is a local maximizer $\hat{\theta }$ such that $∥\hat{\theta }-\theta_{0}∥=O_{p}\left(n^{-1/2}+a_{n}\right)$.*

**Theorem 2.**  *(Oracle Property). Suppose that Assumptions 1−8 hold, and I $\left({\tilde{\beta }}_{0},\gamma_{0}\right)$ is negative definite. If $\gamma_{n}-\gamma_{0}=o_{p}\left(1\right)$ for some $\gamma_{0}>0,\hat{\theta }={\left(\hat{\rho },{\hat{\tilde{\beta }}}_{1}^{T},{\hat{\tilde{\beta }}}_{2}^{T}\right)}^{T}$ must satisfy:*

*(1)*  *sparsity, that is, ${\hat{\tilde{\beta }}}_{n2}=0$ with probability 1;*
*(2)*  *asymptotic normality:*
$$\sqrt{n}\left(I_{1}\left({\tilde{\beta }}_{01},\gamma_{0}\right)+\Sigma_{1}\right)\left\{\left({\hat{\tilde{\beta }}}_{n1}-{\tilde{\beta }}_{01}\right)+{\left(I_{1}\left({\tilde{\beta }}_{01},\gamma_{0}\right)+\Sigma_{1}\right)}^{-1}\Delta \right\}\to N\left(0,\Sigma_{2}\right),$$
*where ${\hat{\tilde{\beta }}}_{n1}={\left(\hat{\rho },{\hat{\tilde{\beta }}}_{11},\ldots ,{\hat{\tilde{\beta }}}_{1s}\right)}^{T}$, and ${\tilde{\beta }}_{01}={\left(\rho_{0},{\tilde{\beta }}_{01},\ldots ,{\tilde{\beta }}_{0s}\right)}^{T}$,*
$$\begin{array}{cc} & \Sigma_{1}=\mathrm{diag}\left\{p_{\lambda_{1}}^{\prime \prime }\left(\left|{\tilde{\beta }}_{01}\right|\right),\ldots ,p_{\lambda_{s}}^{\prime \prime }\left(\left|{\tilde{\beta }}_{0s}\right|\right)\right\}\\ & \Sigma_{2}=\mathrm{cov}\left(\exp\left(-r^{2}/\gamma_{0}\right)\frac{2r}{\gamma_{0}}Z_{i1}\right)\\ & \Delta ={\left(p_{\lambda_{1}}^{\prime }\left(\left|{\tilde{\beta }}_{01}\right|\right)\mathrm{sign}\left({\tilde{\beta }}_{01}\right),\ldots ,p_{\lambda_{s}}^{\prime }\left(\left|{\tilde{\beta }}_{0s}\right|\right)\times \mathrm{sign}\left({\tilde{\beta }}_{0s}\right)\right)}^{T}\\ & I_{1}\left({\tilde{\beta }}_{01},\gamma_{0}\right)\\ & =\frac{2}{\gamma_{0}}E\left[\exp\left(-r^{2}/\gamma_{0}\right)\left(\frac{2r^{2}}{r_{0}}-1\right)\right]\times \left(EZ_{i1}Z_{i1}^{T}\right). \end{array}$$

The detailed proofs of Theorem 1 and Theorem 2 are shown in the Appendix A and Appendix B.

### 2.4. The Selection of Parameter γ

Parameter γ can control the robustness and efficiency of the robust variable selection method. Wang et al., (2013) [7] proposed a parameter selection method based on normal regression. In this paper, we extend the selection method of parameter γ to the spatial Durbin model. The specific process is as follows:

Step 1. Initialize $\hat{\rho }=\rho^{\left(0\right)}$ and $\hat{\tilde{\beta }}={\tilde{\beta }}^{\left(0\right)}$. Set $\rho^{\left(0\right)}=\frac{1}{2},{\tilde{\beta }}^{\left(0\right)}$ a robust estimator. Rewrite the model $Y=\rho WY+X\tilde{\beta }++WX\delta +\epsilon$ as $Y^{*}=X^{*}{\tilde{\beta }}^{*}+\epsilon$, where $Y^{*}=Y-\rho WY$, $X^{*}=\left[X\;WX\right],{\tilde{\beta }}^{*}={\left[\tilde{\beta },\delta \right]}^{T}$.

Step 2. Find the pseudo-outlier set of the sample: Let $D_{n}=\left\{\left(X_{1}^{*},Y_{1}^{*}\right),\ldots ,\left(X_{n}^{*},Y_{n}^{*}\right)\right\}$. Calculate $r_{i}\left(\hat{{\tilde{\beta }}^{*}}\right)=Y_{i}^{*}-X_{i}^{*}\hat{{\tilde{\beta }}^{*}},i=1,\ldots ,n$ and $S_{n}=1.4826\times {\mathrm{median}}_{i}|r_{i}\left({\hat{\tilde{\beta }}}^{*}\right)-$${\mathrm{median}}_{j}\left(r_{j}\left({\hat{\tilde{\beta }}}^{*}\right)\right)|$. Then, there exist the pseudo-outlier set $D_{m}=\left\{\left(X_{i},Y_{i}\right):\left|r_{i}\left({\hat{\tilde{\beta }}}^{*}\right)\right|\geq 2.5S_{n}\right\}$, set $m=♯\left\{1\leq i\leq n:\left|r_{i}\left({\hat{\tilde{\beta }}}^{*}\right)\right|\geq \right.$$\left.2.5S_{n}\right\}$, and $D_{n-m}=D_{n}\setminus D_{m}$.

Step 3. Select the tuning parameter $\gamma_{n}$: construct $\hat{V}\left(\gamma \right)={\left\{\hat{I}\left({\hat{\tilde{\beta }}}^{*}\right)\right\}}^{-1}{\tilde{\Sigma }}_{2}{\left\{\hat{I}\left({\hat{\tilde{\beta }}}^{*}\right)\right\}}^{-1}$, in which

$$\hat{I}\left({\hat{\tilde{\beta }}}^{*}\right)=\frac{2}{\gamma }\left\{\frac{1}{n}\sum_{i=1}^{n}\exp\left(-r_{i}^{2}\left({\hat{\tilde{\beta }}}^{*}\right)/\gamma \right)\left(\frac{2r_{i}\left({\hat{\tilde{\beta }}}^{*}\right)}{\gamma }-1\right)\right\}\cdot \left(\frac{1}{n}\sum_{i=1}^{n}X_{i}X_{i}^{T}\right),$$

$${\tilde{\Sigma }}_{2}=\mathrm{Cov}\left\{\exp\left(-r_{1}^{2}\left({\hat{\tilde{\beta }}}^{*}\right)/\gamma \right)\frac{2r_{1}\left({\hat{\tilde{\beta }}}^{*}\right)}{\gamma }X_{1},\ldots ,\exp\left(-r_{n}^{2}\left({\hat{\tilde{\beta }}}^{*}\right)/\gamma \right)\frac{2r_{n}\left({\hat{\tilde{\beta }}}^{*}\right)}{\gamma }X_{n}\right\}.$$

Next, let $\gamma_{n}$ be the minimizer of $\det(\hat{V}\left(\gamma \right))$ in the set G={γ:ζ(γ)∈(0,1]}, where ζ(·) enjoys the common definition with that in Wang et al., (2013) [7].

Step 4. Update $\hat{\rho }$ and $\hat{\beta }$ as the optimal solution of $\min_{\tilde{\beta }\in R^{p},\rho \in \left[0,1\right]}\frac{1}{n}\sum_{i=1}^{n}\phi_{\gamma }\left(Y_{i}-\rho {\tilde{Y}}_{i}-{\tilde{X}}_{i}\tilde{\beta }\right)$, where $\tilde{Y}=WY$, $\tilde{X}=\left[X\;WX\right]$, $\tilde{\beta }={\left[\beta ,\delta \right]}^{T}$. Go to Step 2 until convergence.

In the above process, the initial step requires an initial value ${\tilde{\beta }}^{\left(0\right)}$. In practice, the estimate of LAD loss is usually used as ${\tilde{\beta }}^{\left(0\right)}$.

### 2.5. The Selection of Parameter λ and $\eta_{j}$

We order $\lambda_{i}=\lambda \cdot \eta_{i}$, in which λ and $\eta_{i}$ are from model (7). Usually, researchers use cross-validation, AIC, and BIC criteria to select $\lambda_{i}$. In this paper, considering the complexity of computation and the consistency of variable selection, we adopt the method of Wang, Li, and Tsai (2007) [16] to consider regularization parameters by minimizing the BIC-type objective function. The BIC-type objective function is as follows:

$$\begin{array}{c} \sum_{t=1}^{n}\left[1-\exp{\left(Y_{i}-\rho {\tilde{Y}}_{i}-{\tilde{X}}_{i}\tilde{\beta }\right)}^{2}/\gamma \right]+n\sum_{j=1}^{2p}\lambda_{i}\left|{\tilde{\beta }}_{j}\right|-\sum_{j=1}^{2p}\log\left(0.5n\lambda_{j}\right)\log\left(n\right), \end{array}$$

The selection method of parameter γ is given above. This makes $\lambda_{i}=\log\left(n\right)/n\left|\theta_{i}\right|$. In practice, let $\theta_{i}={\hat{\theta }}_{i}$, where ${\hat{\theta }}_{i}$ is the exponential square loss estimator without penalty term. Note that this choice satisfies the condition ${\hat{\lambda }}_{i}\to 0$ for $i\leq d_{0}$, and ${\hat{\lambda }}_{i}\to \infty$ for $i>d_{0}$, with $d_{0}$ as the number of nonzero value in the $\theta_{0}$. Therefore, the final estimator can ensure the consistency of variable selection.

## 3. Algorithm for Model Solving

In this section, we focus on designing algorithms to solve model (7). This optimization problem has two optimization variables, $\tilde{\beta }\in R^{2p}$ and ρ∈[0,1]. So, the block coordinate descent algorithm becomes our first choice. However, the subproblems used to solve $\tilde{\beta }$ are nonconvex functions and are not differentiable, and the convergence of the block coordinate drop algorithm is difficult to guarantee. In this case, we used bump decomposition and CCCP algorithms to deal with it. Finally, regarding the processing of penalty terms in the optimization model, we use the ISTA algorithm. This is reflected below.

### 3.1. Block Coordinate Descent Algorithm Frame

We present the framework of the block coordinate descent algorithm in Algorithm 1.    
**Algorithm 1:** Block coordinate descent algorithm1. Set initial value for ${}^{0}\in R^{2p}\;\mathrm{and}\;\rho^{0}\in \left(0,1\right)$;2. repeat {Fork=0,1,2,⋯}3.      Solve the subproblem about ρ with initial point $\rho^{k}$: (8)$$\rho^{k+1}\leftarrow \min_{\rho \in [0,1]}L\left({\tilde{\beta }}^{k},\rho \right)$$4.      Solve the subproblem with initial value ${\tilde{\beta }}^{k}$,
(9)$$\beta^{k+1}\leftarrow \min_{\tilde{\beta }\in R^{2p}}L\left(\tilde{\beta },\rho^{k+1}\right)$$      to obtain a solution ${\tilde{\beta }}^{k+1}$, ensuring that $L\left({\tilde{\beta }}^{k},\rho^{k+1}\right)-L\left({\tilde{\beta }}^{k+1},\rho^{k+1}\right)\leq 0$, and ${\tilde{\beta }}^{k+1}$ is a stationary point of $L\left(\tilde{\beta },\rho^{k+1}\right)$.5. until convergence.

    Next, we need to solve subproblems (8) and (9).

### 3.2. Solving the Subproblem (8)

Subproblem (8) minimizes the univariate function at the interval [0, 1], so it can be solved using a golden section algorithm based on parabolic interpolation. For more information about the algorithm, see Forsythe et al., (1977) [17]. It is not repeated in this article.

### 3.3. Solving the Subproblem (9)

For subproblem (9), by observation, we can see that the penalty term part of the optimization model is the convex function, and the loss function part $\phi_{\gamma }$ can also be decomposed into the difference between the two convex functions, that is, the DC function. So, subproblem (8) is DC programming. We can construct corresponding algorithms to solve the problem.

We can first perform a DC decomposition of the loss function $\phi_{\gamma }\left(t\right)=1-\exp\left(-t^{2}/\gamma \right)$. Suppose there are two convex functions F(t) and G(t), make $F\left(t\right)-G\left(t\right)=\phi_{\gamma }\left(t\right)$. Because $F\left(t\right)=G\left(t\right)+\phi_{\gamma }\left(t\right)$ is a convex function, $F^{\prime \prime }\left(t\right)=\phi_{\gamma }^{\prime \prime }\left(t\right)+G^{\prime \prime }\left(t\right)>0,\;\forall t\in R$. We may as well order $G^{\prime \prime }\left(t\right)=\frac{2}{\gamma }\frac{2}{\gamma }t^{2}$. So, we can make $G\left(t\right)=\frac{1}{3\gamma^{2}}t^{4}$, $F\left(t\right)=G\left(t\right)+\phi_{\gamma }\left(t\right)=1-\exp\left(-t^{2}/\gamma \right)+\frac{1}{3\gamma^{2}}t^{4}$. It can be verified that both F(t) and G(t) are convex functions.

The DC decomposition of $\phi_{\gamma }\left(t\right)$ is as follows:

(10) $$F\left(t\right)=1-\exp\left(-t^{2}/\gamma \right)+\frac{1}{3\gamma^{2}}t^{4},$$

(11) $$G\left(t\right)=\frac{1}{3\gamma^{2}}t^{4},$$

(12) $$\phi_{\gamma }\left(t\right)=F\left(t\right)-G\left(t\right).$$

We can use the CCCP algorithm to solve the problem after DC decomposition. Next, define the following two functions:

(13) $$\begin{array}{cc} \; & J_{\mathrm{vex}}\left(\tilde{\beta }\right)=\frac{1}{n}\sum_{i=1}^{n}F\left(Y_{i}-\rho^{k+1}\langle w_{i},Y\rangle -X_{i}\tilde{\beta }\right)+\lambda \sum_{j=1}^{2p}P\left(\left|{\tilde{\beta }}_{j}\right|\right), \end{array}$$

(14) $$\begin{array}{cc} \; & J_{\mathrm{cav}}\left(\tilde{\beta }\right)=\frac{1}{n}\sum_{i=1}^{n}G\left(Y_{i}-\rho^{k+1}\langle w_{i},Y\rangle -X_{i}\tilde{\beta }\right). \end{array}$$

$w_{i}$ is the *i* th row of the weight matrix *W*, and $\sum_{j=1}^{p}P\left(\left|{\tilde{\beta }}_{j}\right|\right)$ is a convex penalty with respect to $\tilde{\beta }$. Then, $J_{\mathrm{vex}}\left(\cdot \right)$ and $J_{\mathrm{cav}\;}\left(\cdot \right)$ are a convex function and concave function, respectively. So, the suboptimization problem (9) can be rewritten as

(15) $$\min_{\tilde{\beta }\in R^{2p}}L\left(\tilde{\beta },\rho^{k+1}\right)=J_{\mathrm{vex}}\left(\tilde{\beta }\right)+J_{\mathrm{cav}}\left(\tilde{\beta }\right).$$

At this point, it can be found that the optimization problem (15) can be solved by the CCCP(Concave–Convex Procedure) algorithm. The CCCP algorithm framework is shown below (Algorithm 2):    
**Algorithm 2:** The Concave–Convex Procedure (CCCP)1. Initialize ${\tilde{\beta }}^{0}$. Set k=0.2. repeat3.
(16)$${\tilde{\beta }}^{k+1}={\mathrm{argmin}}_{\tilde{\beta }}J_{\mathrm{vex}}\left(\tilde{\beta }\right)+J_{\mathrm{cav}}^{\prime }\left({\tilde{\beta }}^{k}\right)\cdot \tilde{\beta }$$4. untill convergence of ${\tilde{\beta }}^{k}$.

It is easy to know that the optimization problem (16) is a convex optimization problem. The CCCP algorithm minimizes the problem (15) by iteratively solving a series of convex problems (16). Therefore, the solving method of subproblem (16) directly affects the iterative efficiency of the CCCP algorithm.

Observe subproblem (16): $J_{\mathrm{cav}}^{\prime }\left({\tilde{\beta }}^{k}\right)\cdot \tilde{\beta }$ is a linear function about $\tilde{\beta }$. $J_{\mathrm{vex}}\left(\tilde{\beta }\right)$ contains the convex function $\frac{1}{n}\sum_{i=1}^{n}F\left(Y_{i}-\rho^{k+1}\langle w_{i},Y\rangle -X_{i}\tilde{\beta }\right)$ and penalty term $\lambda \sum_{j=1}^{2p}P\left(\left|{\tilde{\beta }}_{j}\right|\right)$ for $\tilde{\beta }$. We might as well order

(17) $$\psi \left(\tilde{\beta }\right)=\frac{1}{n}\sum_{i=1}^{n}F\left(Y_{i}-\rho^{k+1}\langle w_{i},Y\rangle -X_{i}\tilde{\beta }\right)+J_{\mathrm{cav}}^{\prime }\left({\tilde{\beta }}^{k}\right)\cdot \tilde{\beta },$$

where $\psi (\tilde{\beta })$ is a convex function about $\tilde{\beta }$. So, subproblem (16) can be represented as

(18) $$\min_{\tilde{\beta }\in R^{p}}\psi \left(\tilde{\beta }\right)+\lambda \sum_{i=1}^{p}P\left(\left|{\tilde{\beta }}_{i}\right|\right).$$

Optimization problems (17) are composed of convex functions and adaptive lasso penalty terms, and we can use the ISTA algorithm to solve such problems.

For all L>0, ISTA approximates the function $F\left(\beta \right)=\psi \left(\tilde{\beta }\right)+\lambda \sum_{i=1}^{2p}\eta_{i}\left|{\tilde{\beta }}_{i}\right|$ at $\tilde{\beta }=\xi$ as

(19) $$Q_{L}\left(\tilde{\beta },\xi \right)=\psi \left(\xi \right)+\langle \tilde{\beta }-\xi ,\nabla \psi \left(\xi \right)\rangle +\frac{L}{2}{∥\tilde{\beta }-\xi ∥}^{2}+\lambda \sum_{i=1}^{2p}\eta_{i}\left|{\tilde{\beta }}_{i}\right|.$$

This function has the following minimum point:

(20) $$\begin{array}{cc} \Theta_{L}\left(\xi \right) & ={\mathrm{argmin}}_{\tilde{\beta }\in R^{2p}}Q_{L}\left(\tilde{\beta },\xi \right)\\ \; & ={\mathrm{argmin}}_{\tilde{\beta }\in R^{2p}}\left\{\lambda \sum_{i=1}^{2p}\eta_{i}\left|{\tilde{\beta }}_{i}\right|+\frac{L}{2}{∥\tilde{\beta }-\left(\xi -\frac{1}{L}\nabla \psi \left(\xi \right)\right)∥}^{2}\right\}\\ \; & =S_{\lambda \eta /L}\left(\xi -\frac{1}{L}\nabla \psi \left(\xi \right)\right). \end{array}$$

With $\eta =\left[\eta_{1},\ldots ,\eta_{2p}\right]\in R^{2p}$, and for $\nu =\lambda \eta /L\in R_{+}^{p},S_{\alpha }:R^{2p}\to R^{2p}$ the vector-formed soft-thresholding operator $S_{v}\left(\tilde{\beta }\right)=\bar{\tilde{\beta }},\;{\bar{\tilde{\beta }}}_{i}={\left(\left|{\tilde{\beta }}_{i}\right|-v_{i}\right)}_{+}\mathrm{sgn}\left({\tilde{\beta }}_{i}\right),i=1,\ldots ,2p$.

Thus, the solution of problem (11) can be simply expressed as ${\tilde{\beta }}^{k}=\Theta_{L}\left({\tilde{\beta }}^{k-1}\right)$.

In this article, we use the FISTA algorithm with a faster convergence speed than ISTA (Beck and Teboulle, 2009) [18]. The FISTA algorithm framework with backtracking steps is given below (Algorithm 3):
**Algorithm 3:** FISTA with Backtracking Step for solving (17)Require: A,ξ,wλ>0 Ensure: solution $\tilde{\beta }$1: Step 0. Select $L^{0}>0,\eta >1,{\tilde{\beta }}^{0}\in R^{2p}$ Let $\xi^{1}={\tilde{\beta }}^{0},t^{1}=1$2: Step k(k≥1).3: Determine the smallest non-negative integer $i^{k}$ which make $\bar{L}=\eta^{i^{k}}L^{k-1}$ satisfy4:
$$F\left(\Theta_{\bar{L}}\left(\xi^{k}\right)\right)\leq Q_{\bar{L}}\left(\Theta_{\bar{L}}\left(\xi^{k}\right),\xi^{k}\right).$$5: Let $L^{k}=\eta^{i^{k}}L^{k-1}$ according to (19), calculate:6: $\;{\tilde{\beta }}^{k}=\Theta_{L^{k}}\left(\xi^{k}\right)$7: $\;t^{k+1}=\frac{1}{2}\left[1+\sqrt{1+4{\left(t^{k}\right)}^{2}}\right]$8: $\;\xi^{k+1}={\tilde{\beta }}^{k}+\frac{t^{k}-1}{t^{k+1}}\left({\tilde{\beta }}^{k}-{\tilde{\beta }}^{k-1}\right)$9: Output $\tilde{\beta }:={\tilde{\beta }}^{k}$.

So far, we completed the solution of subproblem (9).

## 4. Numerical Examples

We designed five numerical experiments to verify the performance and accuracy of variable selection methods under different conditions. For example, there are abnormal values in dependent variable Y and too many insignificant covariates.

Data generation will be based on model (1). We make the covariance matrix X an n×(q+3) matrix, and the X obeys the (q+3)-dimensional normal distribution, the mean value is zero, and the covariance matrix is $\left(\sigma_{ij}\right)$, where $\sigma_{ij}=0.5^{\left|i-j\right|}$. This means that the number of samples is *n*, the number of significant covariates is 3, and the number of insignificant covariates is *q*. In the following experiments, we set *n* and *q* to n∈{200,360,500} and q∈{5,20,40,60}. For the spatial regression coefficient ρ, in the experiment, we set it to ρ∈{0.2,0.5,0.8}.

We define the spatial weights matrix as a k-diagonal matrix, i.e., a matrix with only the main diagonal and the k-1 skew diagonals around it as element 1, and the other elements as 0. In numerical experiments, we set k = 7.

The regression coefficient β is set to: $\beta =(\beta_{1},\beta_{2},\beta_{3},0_{q})$, where $\left(\beta_{1},\beta_{2},\beta_{3}\right)=\left(3,2,1.5\right)$. The regression coefficient vector of exogenous variables δ is set to: $\delta =(\delta_{1},\delta_{2}.\delta_{3},0_{q})$, where $\left(\delta_{1},\delta_{2}.\delta_{3}\right)=\left(1.5,1.2,1\right)$, and where $0_{q}$ is a zero vector and its dimension is q; this means that the number of 0 elements of β and δ that we set in the experiment is the same, both of which are *q*. The dependent variable *Y* is generated by the model (2).

For the error term, let $\varepsilon ∼N\left(0,\sigma^{2}I_{n}\right),\sigma^{2}$ obeys uniform distribution, and its generation interval is $\left[\sigma_{1}-0.1,\sigma_{1}+0.1\right]$ with $\sigma_{1}\in \left\{1,2\right\}$. Of course, in practice, the observation noise does not completely conform to the Gaussian distribution, and there may be abnormal values in the response. The abnormal values in the response are discussed in Section 4.3.

To reflect the excellence of this model, we also used square loss and LAD to compare with our exponential square loss. To ensure the accuracy of the experiment, we repeated each experiment 100 times. The following results are the results of MSE in the middle of 100 repeated experiments.We express the median of MSE as MedSE.

### 4.1. Nonregular Estimation of Normal Data

In this section, we conduct experiments on the condition that q = 5, the noise is Gaussian noise, and the penalty term is not set for the parameter estimation model. The results are shown in Table 1. Square, Exp, and LAD represent square loss, exponential square loss, and LAD loss, respectively. (1) This shows that Exp, Square, and LAD made estimates of β and δ, which are close to typical values (the means of the true values of beta1, β2, and β3 are 3.0, 2.0, and 1.6, then, the mean sof the true values of $\delta_{1}$, δ2, and δ3 are 2.0, 1.5, and 1.0.). By comparison, the estimated value obtained by the square loss model is the best. (2) For MedSE, the square loss model also performs the best. (3) The three loss functions can give accurate estimates of the spatial autoregressive coefficients ρ.

### 4.2. Nonregular Estimation for High-Dimensional Data

In this subsection, we made q∈{20,40,60}, and the parameter estimation results of the model on normal data with huge sample dimensions are explained. The results are shown in Table 2. It can be found that the estimation of β,δ, and ρ of any model is far less effective than that of q = 5. The results of MedSE are also not satisfactory. Due to the insufficient number of samples, such results can be expected.

### 4.3. Nonregular Estimation of Data with Outliers in Dependent Variable y

In this subsection, we make the error term ϵ obey the mixed Gaussian distribution $\left(1-\xi_{1}\right)\cdot N\left(0,1\right)+\xi_{1}\cdot N\left(10,6^{2}\right)$, where $\xi_{1}\in \left\{0.01,0.05\right\}$. In this case, the observed y will have many outliers. We illustrate the results (Table 3) of the estimated coefficients of β and δ when the observations of y have outliers. (1) For MedSE, unlike the results in Table 1, where y has no outliers, in almost all tests in Table 3, exponential square loss performed the best. (2) By comparison, the estimated values of β and δ obtained by the exponential square loss model are the best. (3) For the estimation of ρ, the exponential square loss is also the best. Therefore, we can conclude that when y has outliers, the SDM based on exponential square loss has good robustness.

### 4.4. Nonregular Estimation of Data with Noise in Spatial Weight Matrix

In this section, we simulate the presence of noise in the spatial weight matrix. We added a minor disturbance term $\epsilon^{{}^{\prime }}$ to each nonzero element in the spatial weight matrix W, where $\epsilon^{{}^{\prime }}∼\left(1-\xi_{2}\right)\cdot N\left(0,0.001\right)+\xi_{2}\cdot N\left(0,1\right),\xi_{2}\in \left\{0.01,0.03,0.05\right\}$, and all the simulated data are generated with ρ=0.5,σ=1. The test results are shown in Table 4. Compared with normal data (Table 1), the MedSE value increased. Additionally, for each loss function, the estimation of β,δ, and ρ also worsens. When the weight matrix has noise, the exponential square loss and LAD loss have good performance. Compared with the square loss, they have more accurate estimates of the parameters and smaller MedSE values. However, it cannot be denied that LAD loss performs better than exponential square loss.

### 4.5. Estimation with Adaptive-l1 Regularizer

We add adaptive L1 regularization to the loss function in this section and conduct experiments. We also record the average number of zero coefficients correctly selected by the model as “Correct” and the average number of nonzero coefficients incorrectly judged by the model as “Incorrect”.

Table 5 shows the results of adaptive lasso regularization on normal data with q = 5. The results show that, under almost all test results, the SDM model with exponential square loss and adaptive lasso cannot only identify more true zero coefficients (“Correct” with exponential square loss model is almost twice as much as that with square loss and LAD loss model) and nearly zero ‘Incorrect’ numbers but also has the best MedSE and accurate estimation of $\hat{\rho }$.

Table 6 shows the results of adaptive lasso regularization on normal data with q∈{20,40,60}. The results show that when there are too many insignificant covariates, the accuracy of the results of the Square loss model with adaptive lasso and the LAD loss model with adaptive lasso decreases significantly. However, the model with adaptive lasso and exponential square loss is still accurate. It can identify more true “Correct” numbers and nearly zero “Incorrect" numbers and has the best MedSE and precise estimation of $\hat{\rho }$.

Table 7 shows the results of estimation with adaptive-l1 regularization when the observations of y have outliers. The results show that, in almost all test results, the exponential square loss model with adaptive L1 has identified more true zero coefficients (“Correct") and, in most cases, has lower MedSE. Compared with the model without regularization term (Table 3), the model with adaptive L1 has a better effect. In the test, the exponential square loss model with adaptive L1 identified at least 8 zero coefficients and, in most cases, determined 10 zero coefficients. For MedSE, the exponential square loss model with adaptive L1 has the smallest MedSE in all cases, except that when n = 500 and 2q = 10, the MedSE in some cases is slightly larger than the LAD loss model with adaptive L1. This shows that the SDM using exponential square loss and adaptive lasso has excellent variable selection ability and strong robustness when the Y observation has outliers.

Table 8 shows the results of adaptive lasso regularization for data that q = 5, rho = 0.5, and spatial weight matrix has noise. For all test results, the exponential square loss with adaptive L1 identifies more zero coefficients than other models (‘Correct’). Compared with the results of the model without regularization term (Table 4), the model with adaptive L1 has a better effect. However, for MedSE, when n = 200, 2q = 10, the exponential square loss with adaptive L1 is the best; when n = 500, 2q = 10, the LAD loss with adaptive L1 is the best; when n = 360, 2q = 10, the LAD loss with adaptive L1 and the exponential square loss with adaptive L1 have little difference. However, since the exponential square loss with adaptive L1 can identify more nonzero coefficients, we believe that the exponential square loss with adaptive L1 is better than the LAD loss with adaptive L1. The results show that when the spatial weight matrix has estimation error, the SDM with exponential square loss and adaptive lasso has excellent variable selection ability and robustness.

## 5. Application of Practical Examples

In this part, we apply the model to actual data to verify the accuracy and efficiency of variable selection and parameter estimation.

We selected a dataset with 211 observations. The dataset describes house sales in the Baltimore area in 1978 and contains home prices and other relevant features. Original data were made available by Robin Dubin [19], Weatherhead School of Management, Case Western Research University, Cleveland, OH. The characteristics of this data are described in Table 9. We mainly study the relationship between price and several other variables. We let the dependent variable be log(PRICE), and the independent variables are NROOM, DWELL, NBATH, PATIO, FIREPL, AC, BMENT, NSTOR, GAR, AGE, CITCOU, LOTSZ, and SQFT.

We set the spatial weight matrix *W* by geographic location relationship. The geographic location can be determined by features *X* and *Y*. The expression for $w_{ij}$ looks like this:

(21) $$w_{ij}=\frac{1}{{\left(X_{i}-X_{j}\right)}^{2}+{\left(Y_{i}-Y_{j}\right)}^{2}}.$$

In addition, we normalize the spatial weights matrix.

Table 10 shows the variable selection results of SDM for square loss, exponential square loss, and LAD loss with adaptive lasso and no penalty. To make variable selection results more intuitive, we designed Table 11. In Table 11, if the model believes that the independent variable has a positive effect on the dependent variable, we mark it as “+”; if the model believes that the independent variable is negatively correlated to the dependent variable, we mark it as “−”; and if the model considers the independent variable not to affect the dependent variable (the absolute value of the parameter estimate is less than 0.001), we do not label it. Additionally, we let the total number of “+” features be **count “+”**; Let the total number of “−” features be **count “−”**; make the total number of all independent variables related to the dependent variable **count**. We can find that the BIC index, with or without regularization, is the lowest exponential square loss. As seen from Table 10 and Table 11, our variable selection method has a smaller BIC index than other variable selection methods and selects fewer independent variables, making the model more accurate and more straightforward. This fully illustrates the excellence of the variable selection method proposed in this paper.

Next, we analyze our regression results. For the variable NROOM, the six models all think it positively correlates with the house price, so the more rooms, the higher the house price. For variables DWELL, EXP+adaptive-l1, Square+adaptive-l1, and LAD+adaptive-l1, it is not considered that they will impact house prices, while EXP+null, Square+null, and LAD+null think that they have a specific positive correlation with housing prices. The three models believe that if it is a detached unit, it will make the house price higher. For the variable NBATH, all models believe it positively correlates with the housing price. Therefore, the more bathrooms, the higher the house price. For the variables PATIO and FIREPL, the models with regularization term are considered independent of house price; however, the model without regularization term believes that it is related to house price, the regression coefficients are very small, and the signs of regression coefficients are different. Therefore, we believe that these two characteristics have little impact on house price. For the variable AC, the model, without adding the with no regularization term, thinks that it is positively related to the house price; that is, the house price with air conditioning is higher than that without air conditioning. For the variable BMENT, except for EXP+adaptive-l1, other models believe it positively relates to the house price; that is, houses with basements tend to have higher prices. For the variable CITCOU, the nonregularized model considers that it positively correlates with the house price; in Baltimore, houses in the city will be more expensive. For the spatial autocorrelation coefficient, the six models’ estimated values are close to 0.5. It can be seen that the rise in house price will lead to an increase in surrounding house prices. Additionally, we can see that NROOM_W, BMENT_W, and CITCOU_W, under the estimation of the six models, all have negative regression coefficients. Therefore, we can know that the spatial regression coefficients of NROOM, BMENT, and CITCOU are negative. As a result, houses with a lot of rooms, houses with basements, and houses in urban areas can have a negative impact on house prices around them. This is also customary. After all, if all the configurations of a house perform well, people will naturally expect more from the houses around them.

## 6. Conclusions

This paper constructs a robust method for SDM variable selection based on adaptive lasso and exponential square loss. We established the “oracle" nature of the proposed estimators. For the nondifferentiable and nonconvex problems when the model is solved, we design the BCD algorithm, DC decomposition, and CCCP algorithm to solve them. Numerical simulations show that our method has good robustness and accuracy when there is noise in the observed data. Additionally, when the spatial weight matrix estimation is inaccurate, our method also has some robustness. In variable selection, our method is significantly better than exponential squared loss and LAD loss, and almost all zero coefficients can be identified in numerical simulations. Taking the housing price dataset of the Baltimore region in 1978 as an example, the excellence and accuracy of the variable selection method of the SDM proposed in this paper are verified. Our analysis demonstrates the difference between our robust variable selection approach and other penalty regression methods, demonstrating the importance of developing robust variable selection methods.

## Figures and Tables

**Table 1:** Nonregular estimation of normal data (q = 5).

	n=200,2q=10				n=360,2q=10				n=500,2q=10		
	Exp	Square	LAD		Exp	Square	LAD		Exp	Square	LAD
ρ=0.8,σ=1											
$\beta_{1}$	3.0904	2.6866	3.2503		3.1335	2.8487	3.0801		2.8084	2.7947	2.9486
$\beta_{2}$	2.0303	1.9449	1.8899		1.9594	1.9498	2.0897		2.0949	2.1354	2.0207
$\beta_{3}$	1.6422	1.4689	1.8394		1.5725	1.5409	1.3781		1.6174	1.7394	1.6394
$\delta_{1}$	1.242	1.3382	1.3069		1.504	1.6117	1.3924		1.4604	1.2156	1.3616
$\delta_{2}$	1.5109	1.3963	1.3582		1.1245	1.132	1.3174		1.1786	1.1139	1.111
$\delta_{3}$	0.8155	1.0711	1.0625		1.1101	1.0575	0.9693		1.0903	1.0092	1.0871
$\hat{\rho }$	0.8001	0.8011	0.7999		0.7999	0.8006	0.7997		0.8002	0.7979	0.7981
MedSE	0.5994	0.4158	0.4693		0.2518	0.2827	0.3432		0.248	0.234	0.3086
ρ=0.5,σ=1											
$\beta_{1}$	3.0854	3.0349	3.0617		3.1254	2.8039	3.0451		2.8058	3.1899	3.2542
$\beta_{2}$	2.0058	2.1532	1.927		1.9556	2.1823	2.2277		2.0986	1.9975	2.0256
$\beta_{3}$	1.6799	1.3788	1.6744		1.5702	1.4268	1.7227		1.6208	1.6813	1.303
$\delta_{1}$	1.2338	1.219	1.7939		1.4848	1.1814	1.3734		1.458	1.4145	1.6612
$\delta_{2}$	1.4943	1.4233	1.5202		1.1322	1.3266	1.3411		1.186	0.9884	1.3373
$\delta_{3}$	0.8849	1.0766	0.5961		1.1036	0.9614	0.8644		1.0966	0.9671	0.9961
$\hat{\rho }$	0.5021	0.4999	0.5		0.5003	0.5	0.5		0.4998	0.4999	0.4999
MedSE	0.6007	0.388	0.4564		0.2808	0.2829	0.3287		0.2452	0.2262	0.2939
ρ=0.2,σ=1											
$\beta_{1}$	3.0072	2.7008	2.7579		3.0283	2.9572	2.9077		2.635	2.8836	3.0321
$\beta_{2}$	1.8903	1.7081	1.93		1.8152	2.081	1.9718		1.8603	2.0794	1.4453
$\beta_{3}$	1.5386	1.4297	1.571		1.4646	1.2788	1.3697		1.4512	1.5486	1.5858
$\delta_{1}$	0.8622	1.2241	0.9667		1.2297	1.2184	1.015		1.0963	1.184	1.1689
$\delta_{2}$	1.4427	1.0584	0.7845		0.8279	0.8104	1.2721		0.7684	0.889	1.0247
$\delta_{3}$	0.6609	0.5715	1.1202		1.0265	0.9351	0.4027		0.7551	0.8819	0.9224
$\hat{\rho }$	0.2417	0.2419	0.2519		0.2271	0.2365	0.2437		0.25	0.2216	0.2317
MedSE	0.9037	0.8134	0.9757		0.5492	0.6286	0.7235		0.9921	0.5287	0.6407
ρ=0.8,σ=2											
$\beta_{1}$	3.0727	2.8882	3.0548		3.2634	2.7795	3.423		2.6808	3.1902	3.0531
$\beta_{2}$	2.1164	1.8141	2.0596		2.0387	1.6757	2.1636		2.1004	1.9817	1.9528
$\beta_{3}$	1.6747	1.3996	1.4515		1.7457	1.7597	1.7635		1.5395	1.3549	1.2795
$\delta_{1}$	0.9807	1.5773	1.6723		1.4112	1.3493	1.5103		1.4831	1.6088	1.5378
$\delta_{2}$	1.7814	0.8604	0.9949		1.0979	1.1257	1.0441		1.1439	1.0058	1.3651
δ_3	0.7301	0.9358	0.8807		1.0707	0.6032	1.0806		1.0423	1.0172	0.8286
$\hat{\rho }$	0.801	0.8045	0.7943		0.7996	0.8042	0.7978		0.7989	0.7989	0.7897
MedSE	1.2058	0.7731	0.9502		0.5131	0.5493	0.6914		0.5536	0.4719	0.5597
ρ=0.5,σ=2											
$\beta_{1}$	3.0762	3.1916	3.1159		3.2325	3.0528	2.8093		2.6731	3.0178	3.0432
$\beta_{2}$	2.0839	2.2408	1.5295		1.8826	1.9422	2.1974		2.1207	2.1175	1.9125
$\beta_{3}$	1.7169	1.384	1.8689		1.6075	1.7206	1.5534		1.5565	1.355	1.2292
$\delta_{1}$	0.9706	1.3618	1.483		1.437	1.5269	1.7395		1.4862	1.3611	1.1453
$\delta_{2}$	1.7802	1.1337	1.2179		1.0509	1.0885	1.4445		1.1825	1.2947	1.3262
$\delta_{3}$	0.8067	1.2863	1.0691		1.2173	0.8635	0.901		1.0777	0.9428	0.9601
$\hat{\rho }$	0.5044	0.5035	0.5		0.5007	0.4996	0.4986		0.4994	0.4973	0.4998
MedSE	1.2459	0.8065	0.9201		0.6033	0.5434	0.6822		0.5387	0.4699	0.5622
ρ=0.2,σ=2											
$\beta_{1}$	2.9838	3.0811	3.0512		3.1253	2.965	2.7197		2.5382	2.8219	2.7438
$\beta_{2}$	1.9525	1.8371	2.3438		1.7215	1.9963	2.2379		1.8893	1.886	1.8351
$\beta_{3}$	1.5477	1.5198	1.2998		1.4741	1.6448	1.0015		1.4075	1.7982	1.7257
$\delta_{1}$	0.5511	1.5501	1.1878		1.1662	1.1816	0.8059		1.1623	1.0545	1.1654
$\delta_{2}$	1.6614	0.7911	1.9069		0.6863	0.9868	1.2406		0.8082	1.0211	1.3427
$\delta_{3}$	0.5739	0.3887	0.1885		1.1021	0.8204	0.8706		0.7875	0.5343	0.8185
$\hat{\rho }$	0.2624	0.2341	0.2342		0.235	0.2307	0.2313		0.2472	0.2349	0.238
MedSE	1.5138	1.1417	1.5123		0.8143	0.816	0.907		1.0921	0.7422	0.8705

**Table 2:** Nonregular estimation for high-dimensional data.

	n=200,2q=40				n=360,2q=80				n=500,2q=120		
	Exp	Square	LAD		Exp	Square	LAD		Exp	Square	LAD
ρ=0.8,σ=1											
$\beta_{1}$	2.9991	2.9355	2.8898		3	3.1579	3.273		2.1076	2.8197	3.0519
$\beta_{2}$	1.87	1.8534	2.148		2.2471	2.097	1.7728		1.4959	2.1326	2.4031
$\beta_{3}$	1.6641	1.7286	1.3997		1.7933	1.4782	1.3566		1.2695	1.5688	1.1838
$\delta_{1}$	1.5449	1.4123	1.2135		1.1743	0.9747	1.6914		0.8435	1.4414	1.6309
$\delta_{2}$	1.1133	1.2998	1.4747		1.193	1.006	0.6772		1.5524	1.0102	1.3473
$\delta_{3}$	1.104	1.0165	0.8516		1.5647	0.6918	0.8134		0.9871	0.8072	0.3043
$\hat{\rho }$	0.7841	0.7976	0.7812		0.7814	0.7626	0.7698		0.7785	0.7921	0.7578
MedSE	1.0389	1.1975	2.4913		3.9024	1.3519	3.216		3.5719	1.4983	3.4753
ρ=0.5,sigma=1											
$\beta_{1}$	2.9674	3.0461	3.0203		2.8981	3.3433	2.8355		3.0614	3.0561	2.7106
$\beta_{2}$	1.9594	1.956	2.2012		2.2222	1.8716	2.041		2.1271	2.0035	2.1597
$\beta_{3}$	1.7014	1.5528	1.4707		1.698	1.5486	1.533		1.4004	1.8345	1.5845
$\delta_{1}$	1.401	1.4171	1.8907		1.4537	1.3244	1.4937		1.4151	1.6572	1.516
$\delta_{2}$	1.3144	1.3097	1.1209		1.3471	1.0562	1.4014		1.2555	1.1235	0.9392
$\delta_{3}$	1.1186	1.0907	0.9799		0.9439	0.977	1.1517		1.3021	1.2148	1.0304
$\hat{\rho }$	0.4984	0.499	0.5		0.5008	0.5004	0.5		0.4997	0.5007	0.5
MedSE	0.7268	0.8361	1.0131		0.846	0.8735	1.0286		1.152	0.9011	1.1143
ρ=0.2,σ=1											
$\beta_{1}$	2.6774	2.7337	2.2269		2.7222	2.6372	2.5639		2.7679	2.625	2.3669
$\beta_{2}$	1.8758	1.5391	2.0274		1.9726	1.4435	1.4924		1.7517	1.8328	1.7334
$\beta_{3}$	1.6073	1.1691	1.5513		1.5515	1.4517	1.9718		1.3588	1.4107	1.5919
$\delta_{1}$	0.6648	0.1578	−0.522		1.3422	0.5994	0.0136		0.4778	0.5541	0.5164
$\delta_{2}$	1.3401	0.5349	0.2749		1.0115	0.1372	0.3757		−0.186	−0.095	−0.533
$\delta_{3}$	0.8527	0.2299	1.1821		0.4795	0.6847	−0.254		0.4057	0.9201	−0.205
$\hat{\rho }$	0.2731	0.368	0.424		0.309	0.3721	0.4449		0.429	0.4203	0.4601
MedSE	1.6545	3.3194	4.2853		2.3481	3.2567	4.9894		5.0342	3.9322	4.9488
ρ=0.8,σ=2											
$\beta_{1}$	3.0412	2.9279	3.2539		3.0001	2.9425	3.3778		3.0661	2.9154	2.9285
$\beta_{2}$	1.8198	1.731	1.3838		2.2004	2.009	1.6173		2.0961	1.7923	1.9155
$\beta_{3}$	1.5695	1.6793	1.9783		1.818	1.7579	2.1066		1.3409	1.8222	1.9951
$\delta_{1}$	1.3762	1.8133	0.6013		1.0067	1.2925	1.0236		1.2358	1.3486	1.1922
$\delta_{2}$	1.4344	1.0001	1.841		1.3286	1.3243	1.0053		1.0631	1.0136	1.148
$\delta_{3}$	0.9648	1.2588	1.7202		1.4715	1.123	1.021		1.629	0.9606	0.9799
$\hat{\rho }$	0.7775	0.7886	0.7847		0.7808	0.7977	0.7196		0.787	0.7941	0.7652
MedSE	1.9747	1.9176	3.2383		4.2531	2.1049	3.9547		2.641	2.139	4.1654
ρ=0.5,σ=2											
$\beta_{1}$	2.9924	3.127	3.3996		2.9071	2.9556	3.2707		3.1224	2.9192	2.7059
$\beta_{2}$	1.937	1.7325	2.2317		2.1674	2.1939	1.9265		2.1034	2.0763	2.0959
$\beta_{3}$	1.6366	1.9284	1.3111		1.7516	1.5356	1.6939		1.329	1.2335	1.5695
$\delta_{1}$	1.1814	1.3155	1.5799		1.3022	1.3435	1.781		1.3229	1.2476	1.5013
$\delta_{2}$	1.6792	1.4335	1.1793		1.5096	1.4914	0.8587		1.1117	1.3711	0.9883
$\delta_{3}$	1.0417	0.9891	1.1182		0.8649	0.8226	1.0424		1.6105	0.9617	1.3937
$\hat{\rho }$	0.4982	0.5005	0.5		0.5013	0.5015	0.5		0.4992	0.4996	0.5
MedSE	1.7761	1.6992	2.0438		1.9405	1.7439	2.1404		2.3691	1.8227	2.2753
ρ=0.8,σ=2											
$\beta_{1}$	2.7126	2.463	3.0218		2.7365	2.6766	2.3112		2.819	2.7498	2.8593
$\beta_{2}$	1.8389	1.7703	1.1025		1.9165	1.625	1.8649		1.7335	1.7638	1.7436
$\beta_{3}$	1.5575	1.302	1.5166		1.5931	1.1868	1.3592		1.299	1.4037	1.2125
$\delta_{1}$	0.5004	0.375	1.4855		1.1763	0.9432	−0.186		0.4655	0.406	0.846
$\delta_{2}$	1.6485	0.5337	−0.864		1.0869	0.1892	−0.044		−0.227	0.1407	−1.145
$\delta_{3}$	0.8129	0.676	−0.697		0.4247	−0.641	0.3875		0.6011	−0.289	0.6946
$\hat{\rho }$	0.2716	0.3598	0.5		0.311	0.355	0.4821		0.4261	0.4147	0.4469
MedSE	2.1955	3.5415	5.0709		2.8997	3.8662	5.2612		5.3588	4.256	5.583

**Table 3:** Nonregular estimation of data with outliers in dependent variable y.

	n=200,2q=10				n=360,2q=10				n=500,2q=10		
	Exp	Square	LAD		Exp	Square	LAD		Exp	Square	LAD
ρ=0.8,σ=1,ξ=0.01											
$\beta_{1}$	3.053	3.333	2.873		3.02	3.237	2.754		2.882	3.102	2.827
$\beta_{2}$	2.213	1.48	1.958		2.125	2.048	1.836		2.126	1.948	1.754
$\beta_{3}$	1.577	1.579	2.046		1.57	1.857	1.908		1.604	1.677	1.757
$\delta_{1}$	1.341	1.983	0.811		1.444	0.966	1.7		1.464	1.165	1.344
$\delta_{2}$	1.311	0.959	1.736		1.127	1.113	1.199		0.962	0.906	1.722
$\delta_{3}$	0.876	1.224	0.644		1.182	1.284	0.752		1.119	0.382	0.954
$\hat{\rho }$	0.801	0.791	0.798		0.8	0.8	0.786		0.794	0.756	0.799
MedSE	0.609	1.866	1.009		0.398	1.392	0.755		0.405	1.268	0.623
ρ=0.5,σ=1,ξ=0.01											
$\beta_{1}$	3.035	3.371	3.049		3.036	3.123	3.179		2.874	3.031	2.972
$\beta_{2}$	2.217	2.19	2.259		2.094	1.672	1.981		2.14	1.687	2.249
$\beta_{3}$	1.561	1.875	1.646		1.555	2.008	1.582		1.699	1.474	1.436
$\delta_{1}$	1.143	1.37	1.623		1.448	1.432	1.227		1.492	1.464	1.264
$\delta_{2}$	1.395	0.621	1.193		1.136	1.642	0.954		1.075	1.472	1.079
$\delta_{3}$	0.929	0.918	0.768		1.119	0.742	1.213		1.186	1.161	1.138
$\hat{\rho }$	0.5	0.497	0.5		0.5	0.499	0.499		0.501	0.5	0.5
MedSE	0.738	1.341	0.84		0.347	1.097	0.627		0.341	0.959	0.511
ρ=0.2,σ=1,ξ=0.01											
$\beta_{1}$	3.032	2.817	2.936		2.961	3.077	3.17		2.7	3.333	2.752
$\beta_{2}$	2.085	2.497	1.757		1.946	1.646	1.802		1.869	1.9	1.853
$\beta_{3}$	1.52	1.224	1.654		1.441	1.817	1.329		1.506	1.614	1.479
$\delta_{1}$	0.882	1.381	1.323		1.216	0.883	1.478		1.151	1.654	1.018
$\delta_{2}$	1.667	1.117	0.84		0.879	0.785	0.688		0.631	1.323	1.034
$\delta_{3}$	0.723	0.49	1.022		0.996	1.207	0.72		0.809	0.618	1.096
$\hat{\rho }$	0.213	0.207	0.222		0.225	0.23	0.223		0.256	0.188	0.232
MedSE	1.091	1.509	1.065		0.556	1.076	0.807		1.061	1.052	0.625
ρ=0.8,σ=1,ξ=0.05											
$\beta_{1}$	2.845	2.064	3.28		2.914	3.493	3.116		2.935	3.857	3.369
$\beta_{2}$	2.228	3.473	1.405		2.148	2.501	1.688		2.109	2.336	1.564
$\beta_{3}$	1.85	1.076	2.271		1.703	0.18	1.86		1.675	2.19	1.487
$\delta_{1}$	1.41	2.957	0.344		1.361	0.522	0.856		1.791	1.181	1.062
$\delta_{2}$	0.863	0.258	2.166		1.05	-0.3	2.015		0.899	0.782	2.052
$\delta_{3}$	0.869	3.323	0.661		1.255	2.546	0.566		0.95	1.568	1.107
$\hat{\rho }$	0.796	0.788	0.782		0.799	0.794	0.793		0.788	0.77	0.789
MedSE	0.984	4.778	2.45		0.716	3.707	1.366		0.835	3.077	1.048
ρ=0.5,σ=1,ξ=0.05											
$\beta_{1}$	2.894	3.636	2.978		2.8	3.655	2.922		2.882	3.027	3.152
$\beta_{2}$	2.169	1.293	2.131		2.177	2.57	2.54		2.149	1.971	2.333
$\beta_{3}$	1.766	0.053	1.419		1.572	1.919	1.607		1.705	2.184	1.673
$\delta_{1}$	1.215	-0.05	0.866		1.436	1.79	1.238		1.729	0.58	0.811
$\delta_{2}$	1.283	1.593	2.262		1.02	0.449	1.381		1.01	1.381	1.316
$\delta_{3}$	0.833	0.489	0.595		1.068	-0.27	0.837		1.042	-0.14	1.032
$\hat{\rho }$	0.497	0.473	0.5		0.499	0.494	0.501		0.499	0.499	0.5
MedSE	0.627	3.878	1.536		0.506	2.989	0.996		0.799	2.467	0.803
ρ=0.2,σ=1,ξ=0.05											
$\beta_{1}$	3.111	2.373	3.24		2.597	3.516	2.797		2.784	3.617	2.885
$\beta_{2}$	2.294	3.955	2.314		2.195	2.032	1.871		2.013	1.844	2.128
$\beta_{3}$	1.791	0.154	1.293		1.335	1.097	1.344		1.589	1.296	1.467
$\delta_{1}$	1.687	2.849	1.613		1.542	2.57	1.161		1.418	1.035	1.627
$\delta_{2}$	1.536	1.503	0.8		0.98	1.2	1.125		0.8	0.872	1.222
$\delta_{3}$	0.805	1.732	1.143		0.884	0.344	0.76		0.934	1.147	1.145
$\hat{\rho }$	0.133	0.034	0.183		0.189	0.088	0.226		0.221	0.174	0.189
MedSE	0.975	3.461	1.326		1.074	2.428	0.822		0.81	2.047	0.67

**Table 4:** Nonregular Estimation of Data with Noise in Spatial Weight Matrix.

	n=200,2q=10				n=360,2q=10				n=500,2q=10		
	Exp	Square	LAD		Exp	Square	LAD		Exp	Square	LAD
ρ=0.5,σ=1,ξ=0.01											
$\beta_{1}$	3.125	3.143	2.909		3.286	2.614	3.142		2.82	2.138	2.895
$\beta_{2}$	1.692	1.89	1.934		1.3	2.39	2.025		2.07	2.367	1.826
$\beta_{3}$	1.919	1.633	1.597		1.68	0.716	1.622		1.651	2.761	1.473
$\delta_{1}$	1.167	0.737	1.612		0.997	1.4	1.418		1.365	0.86	1.452
$\delta_{2}$	1.422	1.235	1.059		−0.28	0.584	1.318		1.247	0.485	1.191
$\delta_{3}$	0.898	0.038	1.076		2.083	1.16	1.273		0.978	0.799	1.164
$\hat{\rho }$	0.501	0.492	0.496		0.501	0.477	0.5		0.5	0.486	0.5
MedSE	0.636	2.596	0.562		2.657	2.623	0.411		0.275	2.591	0.341
ρ=0.5,σ=1,ξ=0.03											
$\beta_{1}$	2.941	1.728	2.955		1.38	2.193	3.15		2.83	2.295	2.963
$\beta_{2}$	1.143	2.575	2.278		0.298	2.322	1.952		1.88	2.292	2.002
$\beta_{3}$	1.771	2.299	1.386		1.195	0.383	1.267		1.867	1.809	1.619
$\delta_{1}$	1.008	−0.63	1.607		0.019	0.211	1.156		0.396	2.348	1.218
$\delta_{2}$	0.605	2.475	1.326		0.255	0.283	0.961		0.974	1.317	0.994
$\delta_{3}$	0.579	0.146	0.922		0.58	0.826	0.921		0.881	0.699	1.136
$\hat{\rho }$	0.503	0.468	0.495		0.503	0.449	0.499		0.494	0.45	0.498
MedSE	1.561	3.925	0.819		3.645	3.922	0.547		1.227	4.972	0.454
ρ=0.5,σ=1,ξ=0.05											
$\beta_{1}$	3.02	1.981	3.046		3.183	2.054	2.849		2.893	2.507	3.187
$\beta_{2}$	1.479	0.857	2.072		1.259	0.636	2.253		1.911	0.865	2.265
$\beta_{3}$	1.978	0.753	0.897		1.721	1.649	1.299		1.827	2.039	1.362
$\delta_{1}$	0.837	0.645	1.349		0.785	0.67	1.362		0.61	0.934	0.962
$\delta_{2}$	1.557	−0.56	1.26		0.551	−0.68	1.275		0.813	1.384	1.135
$\delta_{3}$	−0.23	−0.13	0.379		1.105	0.509	0.536		1.067	−1.56	1.24
$\hat{\rho }$	0.502	0.431	0.489		0.504	0.431	0.493		0.493	0.459	0.491
MedSE	1.922	5.079	1.207		2.191	4.588	0.805		1.034	5.232	0.69

**Table 5:** Estimation with adaptive-l1 regularizer on normal data (q = 5).

	n=200,2q=10				n=360,2q=10				n=500,2q=10		
	Exp	Square	LAD		Exp	Square	LAD		Exp	Square	LAD
ρ=0.8,σ=1											
Correct	10	5.23	5.78		10	5.53	5.61		10	5.61	5.64
Incorrect	0	0	0		0	0	0		0	0	0
$\hat{\rho }$	0.8008	0.8035	0.8011		0.7999	0.8014	0.7982		0.7997	0.7995	0.801
MedSE	0.3747	0.3887	0.4697		0.1468	0.2843	0.3259		0.1316	0.2374	0.2944
ρ=0.5,σ=1											
Correct	10	5.27	5.61		10	5.47	5.74		10	5.69	5.75
Incorrect	0	0	0		0	0	0		0	0	0
$\hat{\rho }$	0.5013	0.5008	0.502		0.5001	0.5003	0.5005		0.4999	0.4997	0.5005
MedSE	0.3575	0.3514	0.4354		0.1342	0.2751	0.3161		0.1207	0.2217	0.2699
ρ=0.2,σ=1											
Correct	10	5.42	5.52		9.98	5.42	5.36		9	5.3	5.46
Incorrect	0	0.05	0.14		0	0.01	0.03		0	0	0
$\hat{\rho }$	0.2351	0.2375	0.2508		0.2257	0.231	0.2426		0.245	0.2265	0.2407
MedSE	0.7905	0.8637	1.0335		0.4758	0.6328	0.7898		0.8372	0.5565	0.6443
ρ=0.8,σ=2											
Correct	10	5.34	5.12		10	5.1	5.42		9	5.17	5.18
Incorrect	0	0	0		6	0	0		0	0	0
$\hat{\rho }$	0.8017	0.7992	0.8087		0.5033	0.7988	0.8018		0.8002	0.8036	0.7986
MedSE	0.8202	0.7826	0.9687		4.5219	0.5524	0.6503		0.2753	0.4729	0.5452
ρ=0.5,σ=2											
Correct	10	5.34	5.21		10	5.4	5.27		9	5.27	5.23
Incorrect	0	0	0		0	0	0		0	0	0
$\hat{\rho }$	0.5034	0.5014	0.4998		0.5005	0.5001	0.5001		0.4997	0.4988	0.4998
MedSE	0.813	0.75	0.9107		0.3039	0.5554	0.6583		0.2723	0.4426	0.5261
ρ=0.2,σ=2											
Correct	8	5.17	5.11		9	5.51	5.29		9	5.31	5.27
Incorrect	0	0.03	0.23		0	0.01	0.02		0	0	0
$\hat{\rho }$	0.2601	0.2382	0.2301		0.2359	0.2241	0.2535		0.2432	0.246	0.246
MedSE	1.3903	1.0942	1.3318		0.6905	0.7508	1.0301		0.8508	0.7031	0.8261

**Table 6:** Estimation with adaptive-l1 regularizer on normal data of high dimension.

	n=200,2q=40				n=360,2q=80				n=500,2q=120		
	Exp	Square	LAD		Exp	Square	LAD		Exp	Square	LAD
ρ=0.8,σ=1											
Correct	40	21.32	21.19		80	42.42	42.63		119.01	65.11	64.21
Incorrect	0	0.02	0.04		0	0	0.03		0	0.02	0.07
$\hat{\rho }$	0.7991	0.8011	0.769		0.8	0.788	0.775		0.7995	0.773	0.773
MedSE	0.1818	1.091	1.746		0.1553	1.348	1.969		0.2672	1.478	1.992
ρ=0.5,σ=1											
Correct	40	21.61	22.4		80	43.52	45.49		119.99	66.78	69.73
Incorrect	0	0	0		0	0	0		0	0	0
$\hat{\rho }$	0.4984	0.5018	0.5		0.5003	0.5	0.5		0.5005	0.5	0.5
MedSE	0.1826	0.8458	0.767		0.1489	0.867	0.788		0.2289	0.915	0.809
ρ=0.2,σ=1											
Correct	39.99	20.74	21.06		73.99	41.98	42.53		109.99	62.69	63.91
Incorrect	0	0.65	0.89		0	0.72	1.15		0.99	0.72	1.14
$\hat{\rho }$	0.2206	0.3554	0.375		0.2476	0.3644	0.437		0.3424	0.371	0.431
MedSE	0.4032	2.9396	3.381		0.8237	3.4479	3.853		2.6921	3.691	3.975
ρ=0.8,σ=2											
Correct	38	20.31	20.9		77.98	41.06	42.05		117.99	62.58	62.78
Incorrect	0	0.02	0.05		0	0.01	0.01		0	0	0.16
$\hat{\rho }$	0.7962	0.7944	0.785		0.8002	0.7937	0.778		0.7982	0.793	0.753
MedSE	0.4685	1.7795	1.959		0.3963	2.0106	2.263		0.7239	2.123	2.766
ρ=0.5,σ=2											
Correct	38.02	20.51	21.13		76.99	41.76	43.31		118.01	63.37	65.18
Incorrect	0	0	0		0	0	0		0	0	0
$\hat{\rho }$	0.4963	0.5009	0.5		0.5006	0.4987	0.5		0.5008	0.499	0.5
MedSE	0.4416	1.6128	1.464		0.3987	1.7193	1.522		0.6623	1.776	1.571
ρ=0.8,σ=2											
Correct	38.99	20.73	20.87		75	41.22	42.04		115	62.43	63.03
Incorrect	0	0.81	1.22		0	0.59	1.05		0	0.8	1.1
$\hat{\rho }$	0.219	0.3583	0.461		0.2383	0.3523	0.434		0.2962	0.413	0.462
MedSE	0.5759	3.656	3.804		0.7593	3.6017	3.887		1.8711	4.392	4.039

**Table 7:** Estimation with adaptive-l1 regularization when the observations of y have outliers.

	n=200,2q=10				n=360,2q=10				n=500,2q=10		
	Exp	Square	LAD		Exp	Square	LAD		Exp	Square	LAD
ρ=0.8,σ=1,ξ=0.01											
Correct	10	5.3	5.47		10	5.05	5.45		9.8	5	5.5
Incorrect	0	0.23	0.01		0	0.07	0		0	0.09	0
$\hat{\rho }$	0.8016	0.7759	0.781		0.7997	0.7978	0.7949		0.7957	0.7606	0.7991
MedSE	0.4001	1.8977	0.5016		0.2229	1.6261	0.3415		0.2547	1.4235	0.287
ρ=0.5,σ=1,ξ=0.01											
Correct	10	5.23	5.53		10	5.11	5.68		10	5.03	5.74
Incorrect	0	0.1	0		0	0.02	0		0	0.01	0
$\hat{\rho }$	0.4999	0.5003	0.5024		0.4997	0.4967	0.4987		0.5004	0.4981	0.4999
MedSE	0.5384	1.4093	0.4443		0.1554	1.2247	0.3282		0.1962	1.1521	0.2811
ρ=0.2,σ=1,ξ=0.01											
Correct	9.9	5.15	5.43		10	5.11	5.44		9.1	5.33	5.62
Incorrect	0	0.17	0.14		0	0.02	0.03		0	0.01	0
$\hat{\rho }$	0.2096	0.2569	0.2543		0.2236	0.2091	0.2362		0.2513	0.2344	0.2358
MedSE	0.7234	1.5711	1.0847		0.4201	1.1447	0.8001		0.857	0.9537	0.6616
ρ=0.8,σ=1,ξ=0.05											
Correct	9	5.56	0.7973		10	5.33	0.7993		8.2	5.23	0.7991
Incorrect	0.2	0.73	5.34		0	0.5	5.43		0	0.42	5.73
$\hat{\rho }$	0.797	0.7892	0		0.7998	0.7954	0		0.7857	0.7892	0
MedSE	0.9265	4.6649	0.4994		0.3901	3.7479	0.3404		0.5873	2.9429	0.2881
ρ=0.5,σ=1,ξ=0.05											
Correct	10	5.31	0.5		9.8	5.24	0.4999		8,2	5.13	0.5002
Incorrect	0.1	0.45	5.34		0	0.23	5.87		0	0.18	5.76
$\hat{\rho }$	0.4969	0.4999	0		0.4991	0.4974	0		0.4994	0.4961	0
MedSE	0.475	3.8028	0.4602		0.2339	2.9176	0.332		0.4473	2.4682	0.2825
ρ=0.8,σ=1,ξ=0.05											
Correct	10	5.15	0.2815		8.2	5.06	0.2357		9.1	5.04	0.2364
Incorrect	0	0.25	5.34		0	0.09	5.47		0	0.03	5.34
$\hat{\rho }$	0.1366	0.1648	0.25		0.1762	0.1159	0.02		0.2152	0.1538	0
MedSE	0.4858	3.1858	1.0803		0.2833	2.4497	0.7324		0.5134	2.1076	0.6375

**Table 8:** Estimation with adaptive-l1 regularization with noisy weighting matrix W.

	n=200,2q=10				n=360,2q=10				n=500,2q=10		
	Exp	Square	LAD		Exp	Square	LAD		Exp	Square	LAD
ρ=0.5,σ=1,ξ=0.01											
Correct	8.1	5.24	5.4		10	5.04	5.45		10	5.11	5.54
Incorrect	0	0.34	0		0	0.41	0		0	0.24	0
$\hat{\rho }$	0.4991	0.4974	0.5		0.5005	0.489	0.4989		0.5	0.4835	0.5001
MedSE	1.1925	2.4503	0.54		0.1897	2.5552	0.3621		0.1557	2.2579	0.2916
ρ=0.5,σ=1,ξ=0.03											
Correct	9.8	5.54	5.4		6.3	5.61	5.3		6.1	5.14	5.76
Incorrect	0	0.87	0		1.1	0.84	0		1.9	0.72	0
$\hat{\rho }$	0.5012	0.4644	0.4996		0.4982	0.4665	0.4998		0.4974	0.476	0.4976
MedSE	0.7048	4.6858	0.6433		1.724	3.5277	0.4495		3.0656	3.7178	0.3789
ρ=0.5,σ=1,ξ=0.05											
Correct	9.96	5.2	5.43		7.02	5.37	5.38		6.02	5.29	5.77
Incorrect	0.04	1.21	0		0	1.2	0		1	1.04	0
$\hat{\rho }$	0.5019	0.4653	0.4993		0.4993	0.4758	0.4953		0.4974	0.4491	0.495
MedSE	0.832	4.9396	0.806		1.3822	4.3962	0.5848		2.2125	4.0998	0.4734

**Table 9:** Variable description.

Variable	Description
*STATION*	ID variable
*PRICE*	sales price of house iin $1000 (MLS)
*NROOM*	the number of rooms
*DWELL*	1 if detached unit, 0 otherwise
*NBATH*	the number of bathrooms
*PATIO*	1 if patio, 0 otherwise
*FIREPL*	1 if fireplace, 0 otherwise
*AC*	1 if air conditioning, 0 otherwise
*BMENT*	1 if basement, 0 otherwise
*NSTOR*	number of stories
*GAR*	number of car spaces in garage (0 = no garage)
*AGE*	age of dwelling in years
*CITCOU*	1 if dwelling is in Baltimore County, 0 otherwise
*LOTSZ*	lot size in hundreds of square feet
*SQFT*	interior living space in hundreds of square feet
*X*	x coordinate on the Maryland grid
*Y*	y coordinate on the Maryland grid

**Table 10:** Variable section on real data.

	EXP			Square			LAD	
	**Adaptive-l1**	**Null**		**Adaptive-l1**	**Null**		**Adaptive-l1**	**Null**
NROOM	0.49674002	0.20409727		0.01051881	0.1929546		0.0037123	0.2362159
DWELL	−1.3922 × 10^−17^	0.45980677		0.00075831	0.4703206		0.00029162	0.5097926
NBATH	0.030063578	0.36030577		0.00385469	0.3514846		0.0012552	0.4254525
PATIO	4.91478 × 10^−18^	0.01072777		0.00097357	0.017285		0.00014135	−0.092123
FIREPL	−9.5477 × 10^−18^	−0.01059		0.0002972	0.0013726		0.00012271	−0.077913
AC	−1.2919 × 10^−17^	0.3021609		0.001	0.311554		0.00020267	0.3138447
BMENT	−2.2645 × 10^−17^	0.1187834		0.0044111	0.1235361		0.00116474	0.1317025
NSTOR	−9.9947 × 10^−17^	0.47809045		0.00359286	0.503778		0.00129079	0.4164672
GAR	−2.2383 × 10^−17^	−0.1040092		0.00038606	−0.099652		0.00039553	−0.0844
AGE	4.72988 × 10^−17^	0.01105389		0.03319136	0.0113282		0.02091404	0.0100436
CITCOU	−6.0274 × 10^−17^	0.68202393		0.00093599	0.6868509		0.00025428	0.4701451
LOTSZ	1.04997 × 10^−17^	0.0011463		0.002195	0.0011849		0.00443912	4.606×10^−5^
SQFT	−7.0764 × 10^−17^	−0.0362982		0.0316769	−0.037256		0.01075005	−0.034887
NROOM_W	−0.28507527	−0.1092328		−0.012775	−0.098775		−0.0059391	−0.17384
DWELL_W	1.61355 × 10^−33^	−0.1051101		−0.0010685	−0.102124		−0.0005411	−0.107508
NBATH_W	−7.3257 × 10^−18^	−0.1575366		−0.0033218	−0.159341		−0.0014154	−0.095605
PATIO_W	−8.7577 × 10^−18^	0.0642994		−8.43 × 10^−5^	0.0601125		1.1236 × 10^−5^	0.1341514
FIREPL_W	−4.6005 × 10^−34^	−0.0387668		0.00068287	−0.036572		−2.587 × 10^−5^	−0.042569
AC_W	−3.3933 × 10^−17^	−0.2098427		−0.001	−0.223255		−0.0004486	−0.187274
BMENT_W	−0.02780421	−0.111448		−0.0074127	−0.117315		−0.0032221	−0.1317
NSTOR_W	−2.6009 × 10^−32^	−0.1648658		−0.0047893	−0.159493		−0.0023109	−0.178134
GAR_W	1.94949 × 10^−17^	0.15495116		0.001	0.1566788		0.00017173	0.1186948
AGE_W	5.87444 × 10^−33^	−0.0069188		−0.0228056	−0.007876		−0.0204497	−0.001951
CITCOU_W	−0.06178084	−0.4116914		−0.0030172	−0.426973		−0.001	−0.240381
LOTSZ_W	−2.2196 × 10^−32^	−0.0005826		−0.0034412	−0.000541		−0.0057558	−0.000449
SQFT_W	2.75575 × 10^−17^	0.01072435		−0.0207167	0.0098465		−0.0112853	0.0143746
ρ	0.498613719	0.49970041		0.49571237	0.4997043		0.49780529	0.499992
MSE	0.121911727	0.11475312		0.13792606	0.1149259		0.14467343	0.114829
BIC	−304.892336	−317.66083		−278.85061	−317.3434		−268.77299	−317.5214

**Table 11:** Visual representation of variable selection on real data.

	EXP			Square			LAD	
	**Adaptive-l1**	**Null**		**Adaptive-l1**	**Null**		**Adaptive-l1**	**Null**
NROOM	**+**	**+**		**+**	**+**		**+**	**+**
DWELL		**+**			**+**			**+**
NBATH	**+**	**+**		**+**	**+**		**+**	**+**
PATIO		**+**			**+**			**−**
FIREPL		**−**			**+**			**−**
AC		**+**			**+**			**+**
BMENT		**+**		**+**	**+**		**+**	**+**
NSTOR		**+**		**+**	**+**		**+**	**+**
GAR		**−**			**−**			**−**
AGE		**+**		**+**	**+**		**+**	**+**
CITCOU		**+**			**+**			**+**
LOTSZ		**+**		**+**	**+**		**+**	
SQFT		**−**		**+**	**−**		**+**	**−**
NROOM_W	**−**	**−**		**−**	**−**		**−**	**−**
DWELL_W		**−**		**−**	**−**			**−**
NBATH_W		**−**		**−**	**−**		**−**	**−**
PATIO_W		**+**			**+**			**+**
FIREPL_W		**−**			**−**			**−**
AC_W		**−**			**−**			**−**
BMENT_W	**−**	**−**		**−**	**−**		**−**	**−**
NSTOR_W		**−**		**−**	**−**		**−**	**−**
GAR_W		**+**			**+**			**+**
AGE_W		**−**		**−**	**−**		**−**	**−**
CITCOU_W	**−**	**−**		**−**	**−**			**−**
LOTSZ_W				**−**			**−**	
SQFT_W		**+**		**−**	**+**		**−**	**+**
count “+”	2	13		7	14		7	11
count “−”	3	12		9	11		7	13
count	5	25		16	25		14	24
BIC	−304.892336	−317.66083		−278.85061	−317.3434		−268.77299	−317.5214

## Data Availability

Not applicable.

## Appendix A. Proof of Theorem 1

Let $\xi_{n}=n^{-1/2}+a_{n}$ and set ∥u∥=C, where u is *d*-dimensional vector and *C* is a large enough constant. Similar to Fan and Li (2001), we first show that $∥\hat{\tilde{\beta }}-{\tilde{\beta }}_{0}∥=O_{p}\left(\xi_{n}\right)$. It suffices to show that, for any given ϵ>0, there is a large constant *C* such that, for large *n*,

(A1) $$P\left\{\underset{|u|=C}{\sup}\ell_{n}\left(\theta_{0}+\xi_{n}u\right)<\ell_{n}\left(\theta_{0}\right)\right\}\geq 1-\epsilon .$$

Define $Z={\left(I-\rho W\right)}^{-1}{\tilde{X}}^{T}$, $\epsilon^{*}={\left(I-/rhoW\right)}^{-1}\epsilon_{n}$, and then we can represent model (1) as

(A2) $$Y={\left(I-\rho W\right)}^{-1}{\tilde{X}}^{T}\tilde{\beta }+{\left(I-\rho W\right)}^{-1}\varepsilon =Z^{T}\beta +\varepsilon^{*}.$$

For the optimization model (7)

$$\min_{\tilde{\beta }\in R^{2p},\rho \in \left[0,1\right]}L\left(\tilde{\beta },\rho \right),$$

we know that this is equivalent to

$$\max_{\tilde{\tilde{\beta }}\in R^{2p},\rho \in \left[0,1\right]}-L\left(\tilde{\beta },\rho \right),$$

which can be expressed as

(A3) $$\ell_{n}\left(\theta \right)=\sum_{i=1}^{n}\exp\left\{-\left(Y_{i}-Z_{i}\tilde{\beta }\right)/\gamma_{n}\right\}-n\sum_{j=1}^{2p}p_{\lambda_{j}}\left(\left|{\tilde{\beta }}_{j}\right|\right).$$

Let $D_{n}\left(\theta ,\gamma \right)=\sum_{i=1}^{n}\exp\left\{-{\left(Y_{i}-Z_{i}\tilde{\beta }\right)}^{2}/\gamma \right\}\frac{2\left(Y_{i}-Z_{i}\tilde{\beta }\right)}{\gamma }Z_{i}$. Since $p_{\lambda_{j}}\left(0\right)=0$ for j=1,2,…,p, we have

(A4) $$\begin{array}{cc} \; & \ell_{n}\left(\theta_{0}+\xi_{n}u\right)-\ell_{n}\left(\theta_{0}\right)\\ \; & =\sum_{i=1}^{n}\exp\left\{-\left(Y_{i}-Z_{i}\left({\tilde{\beta }}_{0}+\xi_{n}u\right)\right)/\gamma_{n}\right\}-\sum_{i=1}^{n}\exp\left\{-\left(Y_{i}-Z_{i}{\tilde{\beta }}_{0}\right)/\gamma_{n}\right\}-\sum_{j=1}^{2p}\left\{p_{\lambda_{j}}\left(\left|{\tilde{\beta }}_{j0}+\xi_{nu_{j}}\right|\right)-p_{\lambda_{j}}\left(\left|{\tilde{\beta }}_{j0}\right|\right)\right\}\\ \; & \leq \sum_{i=1}^{n}\exp\left\{-\left(Y_{i}-Z_{i}\left({\tilde{\beta }}_{0}+\xi_{n}u\right)\right)/\gamma_{n}\right\}-\sum_{i=1}^{n}\exp\left\{-\left(Y_{i}-Z_{i}{\tilde{\beta }}_{0}\right)/\gamma_{n}\right\}-\sum_{j=1}^{s}\left\{p_{\lambda_{j}}\left(\left|{\tilde{\beta }}_{j0}+\xi_{nu_{j}}\right|\right)-p_{\lambda_{j}}\left(\left|{\tilde{\beta }}_{j0}\right|\right)\right\}\\ \; & =S_{n}\left(u\right)+K_{n}\left(u\right). \end{array}$$

Note that

(A5) $$\begin{array}{cc} \; & S_{n}\left(∥u∥\right)=\sum_{i=1}^{n}\exp\left\{-\frac{{\left(Y_{i}-Z_{i}\left({\tilde{\beta }}_{0}+\xi_{n}u\right)\right)}^{2}}{\gamma_{n}}\right\}-\sum_{i=1}^{n}\exp\left\{-\frac{{\left(Y_{i}-Z_{i}{\tilde{\beta }}_{0}\right)}^{2}}{\gamma_{n}}\right\}\\ \; & =\xi_{n}\sum_{i=1}^{n}{\left\{\exp\left\{-\frac{{\left(Y_{i}-Z_{i}{\tilde{\beta }}_{0}\right)}^{2}}{\gamma_{n}}\right\}\frac{2\left(Y_{i}-Z_{i}\tilde{\beta }\right)}{\gamma_{n}}Z_{i}^{T}\right\}}^{T}u-\frac{1}{2}u^{T}\left\{-\frac{2}{\gamma_{n}}\int ZZ^{T}e^{-{\left(Y-Z{\tilde{\beta }}_{0}\right)}^{2}/\gamma_{n}}\right.\\ \; & \left.\times \left(\frac{2{\left(Y-Z{\tilde{\beta }}_{0}\right)}^{2}}{\gamma_{n}}-1\right)dF\left(Z,y\right)\right\}un\xi_{n}^{2}\left\{1+o_{p}\left(1\right)\right\}\\ \; & =\xi_{n}D_{n}{\left(\beta_{0},\gamma_{n}\right)}^{T}u-\frac{1}{2}u^{T}\left\{-I\left({\tilde{\beta }}_{0},\gamma_{n}\right)\right\}un\xi_{n}^{2}\left\{1+o_{p}\left(1\right)\right\}. \end{array}$$

Additionally,

(A6) $$\begin{array}{cc} K_{n}\left(u\right) & =n\sum_{j=0}^{s}\left\{p_{\lambda_{j}}\left(\left|{\tilde{\beta }}_{j0}+\xi_{n}u_{j}\right|\right)-p_{\lambda_{j}}\left(\left|{\tilde{\beta }}_{j0}\right|\right)\right\}\\ & =n\xi_{n}\sum_{j=0}^{s}p_{\lambda_{j}}^{\prime }\left(\left|{\tilde{\beta }}_{j0}\right|\right)\mathrm{sign}\left({\tilde{\beta }}_{j0}\right)u_{j}+n\xi_{n}^{2}\sum_{j=0}^{s}p_{\lambda_{j}}^{\prime \prime }\left(\left|{\tilde{\beta }}_{j0}\right|\right)u_{j}^{2}\left\{1+o\left(1\right)\right\}\\ \; & \leq a_{n}n\xi_{n}\sum_{j=0}^{s}\left|u_{j}\right|+b_{n}n\xi_{n}^{2}\sum_{j=0}^{s}u_{j}^{2}\left\{1+o\left(1\right)\right\}\leq a_{n}n\xi_{n}\sum_{j=0}^{s}\left|u_{j}\right|+2b_{n}n\xi_{n}^{2}{∥u∥}^{2}\\ \; & \leq \sqrt{s}a_{n}n\xi_{n}\sum_{j=0}^{s}\left|u_{j}\right|+b_{n}n\xi_{n}^{2}{∥u∥}^{2}. \end{array}$$

Since $\gamma_{n}-\gamma_{0}=o_{p}\left(1\right)$, by Taylor’s expansion, we have

(A7) $$\begin{array}{cc} \; & \ell_{n}\left(\theta_{0}+\xi_{n}u\right)-\ell_{n}\left(\theta_{0}\right)\\ \; & \leq \xi_{n}D_{n}{\left(\theta_{0},\gamma_{n}\right)}^{T}u-\frac{1}{2}u^{T}\left[-I\left(\theta_{0},\gamma_{n}\right)\right]un\xi_{n}^{2}\left\{1+o_{p}\left(1\right)\right\}-\sqrt{s}a_{n}n\xi_{n}\sum_{j=0}^{s}\left|u_{j}\right|+b_{n}n\xi_{n}^{2}{∥u∥}^{2}. \end{array}$$

Note that $n^{-1/2}D_{n}\left(\theta_{0},\gamma_{0}\right)=O_{P}\left(1\right)$. So, there is $O_{p}\left(n^{1/2}\xi_{n}\right)=O_{p}\left(n\xi_{n}^{2}\right)$ in the last equation of (A.7). By choosing a sufficiently large *C*, the second term dominates the first term uniformly in ∥u∥=C. Since $b_{n}=o_{p}\left(1\right)$, the third term is also dominated by the second term of (A.7). Therefore, (A.1) holds by choosing a sufficiently large *C*.

## Appendix B. Proof of Theorem 2

### Appendix B.1. Proof of Theorem 2(i)

Here, we show the proof of the first point of Theorem 2. For this, we need only prove that, as n→∞, there is any beta1 satisfying ${\tilde{\beta }}_{1}-{\tilde{\beta }}_{01}=O_{p}\left(n^{-1/2}\right)$, and for some small $\epsilon_{n}={\mathrm{Cn}}^{-1/2}$ and j=s+1,…,p, we have

(A8) $$\frac{\partial \ell_{n}\left(\tilde{\beta }\right)}{\partial {\tilde{\beta }}_{j}}=\left\{\begin{array}{cc} >0, & \;\mathrm{for}\;0<{\tilde{\beta }}_{j}<\epsilon_{n}\\ <0, & \;\mathrm{for}\;-\epsilon_{n}<{\tilde{\beta }}_{j}<0 \end{array}\right..$$

First, let us make

(A9) $$Q_{n}\left(\tilde{\beta },\gamma \right)=\sum_{i=1}^{n}\exp\left\{-{\left(Y_{i}-Z_{i}^{T}\tilde{\beta }\right)}^{2}/\gamma \right\}.$$

Then,

(A10) $$\frac{\partial \ell_{n}\left(\tilde{\beta }\right)}{\partial {\tilde{\beta }}_{j}}=\frac{\partial Q_{n}\left(\tilde{\beta },\gamma_{n}\right)}{\partial {\tilde{\beta }}_{j}}-np_{\lambda_{j}}^{\prime }\left(\left|{\tilde{\beta }}_{j}\right|\right)\mathrm{sign}\left({\tilde{\beta }}_{j}\right).$$

By Taylor expansion, we can obtain

(A11) $$\begin{array}{cc} \frac{\partial \ell_{n}\left(\tilde{\beta }\right)}{\partial {\tilde{\beta }}_{j}}= & \frac{\partial Q_{n}\left({\tilde{\beta }}_{0},\gamma_{n}\right)}{\partial {\tilde{\beta }}_{j}}+\sum_{l=1}^{p}\frac{\partial^{2}Q_{n}\left({\tilde{\beta }}_{0},\gamma_{n}\right)}{\partial {\tilde{\beta }}_{j}\partial {\tilde{\beta }}_{l}}\left({\tilde{\beta }}_{l}-{\tilde{\beta }}_{l0}\right)+\sum_{l=1}^{p}\sum_{k=1}^{p}\frac{\partial^{3}Q_{n}\left({\tilde{\beta }}^{*},\gamma_{n}\right)}{\partial {\tilde{\beta }}_{j}\partial {\tilde{\beta }}_{l}\partial {\tilde{\beta }}_{k}}\left({\tilde{\beta }}_{l}-{\tilde{\beta }}_{l0}\right)\left({\tilde{\beta }}_{k}-{\tilde{\beta }}_{k0}\right)\\ \; & -np_{\lambda_{j}}^{\prime }\left(\left|{\tilde{\beta }}_{j}\right|\right)\mathrm{sign}\left({\tilde{\beta }}_{j}\right)\\ \; & =R_{11}+R_{12}+R_{13}-np_{\lambda_{j}}^{\prime }\left(\left|{\tilde{\beta }}_{j}\right|\right)\mathrm{sign}\left({\tilde{\beta }}_{j}\right). \end{array}$$

where ${\tilde{\beta }}^{*}$ lies between $\tilde{\beta }$ and ${\tilde{\beta }}_{0}$. Moreover, because

$$n^{-1}\frac{\partial^{2}Q_{n}\left({\tilde{\beta }}_{0},\gamma_{0}\right)}{\partial {\tilde{\beta }}_{j}\partial {\tilde{\beta }}_{l}}=E\left\{\frac{\partial^{2}Q_{n}\left({\tilde{\beta }}_{0}\right)}{\partial {\tilde{\beta }}_{j}\partial {\tilde{\beta }}_{l}}\right\}+o_{p}\left(1\right),$$

$$n^{-1}\frac{\partial Q_{n}\left({\tilde{\beta }}_{0},\gamma_{0}\right)}{\partial {\tilde{\beta }}_{j}}=O_{p}\left(n^{-1/2}\right).$$

So there is $R_{11}=O_{p}\left(\sqrt{n}\right),R_{12}=O_{p}\left(\sqrt{n}\right),$ and $R_{13}=O_{p}\left(\sqrt{n}\right)$. Additionally, because of $b_{n}=o_{p}\left(1\right)$ and $\sqrt{n}a_{n}=o_{p}\left(1\right)$, we are able to make $\tilde{\beta }-{\tilde{\beta }}_{0}=O_{p}\left(n^{-1/2}\right)$.

Since $1/\min_{s+1\leq j\leq d}\left(\sqrt{n}\lambda_{j}\right)=o_{p}\left(1\right)$ and $\lim_{n\to \infty }\inf\lim_{t\to 0+}$$\inf\left\{\min_{s+1\leq j\leq d}p_{\lambda_{j}}\left(|t|\right)/\lambda_{j}\right\}>0$ with probability 1, the sign of the derivative is completely determined by that of $\beta_{j}$. This completes the proof of Theorem 1 (i).

### Appendix B.2. Proof of Theorem 2(ii)

Here, we show the proof of the second point of Theorem 2. For brevity, let ${\tilde{\beta }}_{10}^{*}=\rho$ and ${\tilde{\beta }}_{1j}^{*}={\tilde{\beta }}_{1j},j=1,\ldots ,s,$ then denote ${\tilde{\beta }}_{1}^{*}={\left(\rho ,{\tilde{\beta }}_{11},\ldots ,{\tilde{\beta }}_{1s}\right)}^{T}$ and ${\tilde{\beta }}_{0}^{*}={\left(\rho_{0},{\tilde{\beta }}_{10},\ldots ,{\tilde{\beta }}_{0s}\right)}^{T}$. We known that $\hat{\theta }$ minimizes $Q_{n}\left(\theta \right)$. We showed that there exists a $\sqrt{n}$-consistent local maximizer of $\ell_{n}\left\{\left({\tilde{\beta }}_{1},0\right)\right\}$. satisfying that

$$\frac{\partial \ell_{n}\left\{\left({\hat{\tilde{\beta }}}_{1},0\right)\right\}}{\partial {\tilde{\beta }}_{j}}=0,\;\;\mathrm{for}\;j=1,\ldots ,s.$$

Since ${\hat{\tilde{\beta }}}_{1}$ is a consistent estimator, we have

(A12) $$\begin{array}{cc} \frac{\partial Q_{n}\left\{\left({\hat{\tilde{\beta }}}_{1},0\right),\gamma_{n}\right\}}{\partial \beta_{j}} & -np_{\lambda_{j}}^{\prime }\left(\left|{\hat{\tilde{\beta }}}_{j}\right|\right)\mathrm{sign}\left({\hat{\tilde{\beta }}}_{j}\right)\\ = & \frac{\partial Q_{n}\left({\tilde{\beta }}_{0},\gamma_{n}\right)}{\partial {\tilde{\beta }}_{j}}+\sum_{l=1}^{s}\left\{\frac{\partial^{2}Q_{n}\left({\tilde{\beta }}_{0},\gamma_{n}\right)}{\partial {\tilde{\beta }}_{j}\partial {\tilde{\beta }}_{l}}+o_{p}\left(1\right)\right\}\left({\hat{\tilde{\beta }}}_{l}-{\tilde{\beta }}_{01}\right)\\ \; & -n\left[p_{\lambda_{j}}^{\prime }\left(\left|{\tilde{\beta }}_{0j}\right|\right)\mathrm{sign}\left({\tilde{\beta }}_{0j}\right)+\left\{p_{\lambda_{j}}^{\prime \prime }\left(\left|\beta_{0j}\right|\right)+o_{p}\left(1\right)\right\}\left({\hat{\tilde{\beta }}}_{j}-{\tilde{\beta }}_{0j}\right)\right]=0. \end{array}$$

The above equation can be rewritten as follows:

(A13) $$\frac{\partial Q_{n}\left({\tilde{\beta }}_{0},\gamma_{n}\right)}{\partial {\tilde{\beta }}_{j}}=\sum_{l=1}^{s}\left\{E\left\{\frac{\partial^{2}Q_{n}\left({\tilde{\beta }}_{0},\gamma_{n}\right)}{\partial {\tilde{\beta }}_{j}\partial {\tilde{\beta }}_{l}}\right\}+o_{p}\left(1\right)\right\}\left({\hat{\tilde{\beta }}}_{l}-{\tilde{\beta }}_{01}\right)+n\Delta +n\left[\Sigma_{1}+O_{p}\left(1\right)\right]\left({\hat{\tilde{\beta }}}_{n1}-{\tilde{\beta }}_{01}\right),$$

(A14) $$\begin{array}{cc} \; & nI_{1}\left({\tilde{\beta }}_{01},\gamma_{0}\right)\left({\hat{\tilde{\beta }}}_{n1}-{\tilde{\beta }}_{01}\right)+n\Delta +n\left[\Sigma_{1}+O_{p}\left(1\right)\right]\left({\hat{\tilde{\beta }}}_{n1}-{\tilde{\beta }}_{01}\right)\\ \; & =n\left[I_{1}\left({\tilde{\beta }}_{01},\gamma_{0}\right)+\Sigma_{1}\right]\left({\hat{\tilde{\beta }}}_{n1}-{\tilde{\beta }}_{01}\right)+n\Delta \\ \; & =n\left[I_{1}\left({\tilde{\beta }}_{01},\gamma_{0}\right)+\Sigma_{1}\right]\left({\hat{\tilde{\beta }}}_{n1}-{\tilde{\beta }}_{01}\right)+n\left[I_{1}\left({\tilde{\beta }}_{01},\gamma_{0}\right)+\Sigma_{1}\right]{\left[I_{1}\left({\tilde{\beta }}_{01},\gamma_{0}\right)+\Sigma_{1}\right]}^{-1}\Delta \\ \; & =n\left[I_{1}\left({\tilde{\beta }}_{01},\gamma_{0}\right)+\Sigma_{1}\right]\left\{\left({\hat{\tilde{\beta }}}_{n1}-{\tilde{\beta }}_{01}\right)+n{\left[I_{1}\left({\tilde{\beta }}_{01},\gamma_{0}\right)+\Sigma_{1}\right]}^{-1}\Delta \right\}\\ \; & =-\frac{\partial Q_{n}\left({\tilde{\beta }}_{0},\gamma_{n}\right)}{\partial {\tilde{\beta }}_{j}}+o_{p}\left(1\right). \end{array}$$

Since $\sqrt{n}\left(\gamma_{n}-\gamma_{0}\right)=o_{p}\left(1\right)$, invoking Slutsky’s lemma and the Lindeberg–Feller central limit theorem, we have

(A15) $$\begin{array}{c} \sqrt{n}\left(I_{1}\left({\tilde{\beta }}_{01},\gamma_{0}\right)+\Sigma_{1}\right)\left\{\left({\hat{\tilde{\beta }}}_{n1}-{\tilde{\beta }}_{01}\right)+{\left(I_{1}\left({\tilde{\beta }}_{01},\gamma_{0}\right)+\Sigma_{1}\right)}^{-1}\Delta \right\}\to N\left(0,\Sigma_{2}\right),\\ where\;{\hat{\tilde{\beta }}}_{n1}={\left(\hat{\rho },{\hat{\tilde{\beta }}}_{11},\ldots ,{\hat{\tilde{\beta }}}_{15}\right)}^{T},and\;{\tilde{\beta }}_{01}={\left(\rho_{0},{\tilde{\beta }}_{01},\ldots ,{\tilde{\beta }}_{0s}\right)}^{T},\\ \Sigma_{1}=\mathrm{diag}\left\{p_{\lambda_{1}}^{\prime \prime }\left(\left|{\tilde{\beta }}_{01}\right|\right),\ldots ,p_{\lambda_{s}}^{\prime \prime }\left(\left|{\tilde{\beta }}_{0s}\right|\right)\right\},\Sigma_{2}=\mathrm{cov}\left(\exp\left(-r^{2}/\gamma_{0}\right)\frac{2r}{\gamma_{0}}Z_{i1}\right),\\ \Delta ={\left(p_{\lambda_{1}}^{\prime }\left(\left|{\tilde{\beta }}_{01}\right|\right)\mathrm{sign}\left({\tilde{\beta }}_{01}\right),\ldots ,p_{\lambda_{s}}^{\prime }\left(\left|{\tilde{\beta }}_{0s}\right|\right)\times \mathrm{sign}\left({\tilde{\beta }}_{0s}\right)\right)}^{T},\\ I_{1}\left({\tilde{\beta }}_{01},\gamma_{0}\right)=\frac{2}{\gamma_{0}}E\left[\exp\left(-r^{2}/\gamma_{0}\right)\left(\frac{2r^{2}}{\gamma_{0}}-1\right)\right]\times \left(EZ_{i1}Z_{i1}^{T}\right). \end{array}$$

Then, the proof of part (ii) is completed.
